# From Reef to Table: Social and Ecological Factors Affecting Coral Reef Fisheries, Artisanal Seafood Supply Chains, and Seafood Security

**DOI:** 10.1371/journal.pone.0123856

**Published:** 2015-08-05

**Authors:** John N. Kittinger, Lida T. Teneva, Haruko Koike, Kostantinos A. Stamoulis, Daniela S. Kittinger, Kirsten L. L. Oleson, Eric Conklin, Mahana Gomes, Bart Wilcox, Alan M. Friedlander

**Affiliations:** 1 Conservation International, Betty and Gordon Moore Center for Science and Oceans, 7192 Kalaniana‘ole Hwy, Honolulu, Hawaii, United States of America; 2 Center for Ocean Solutions, Stanford University, Stanford Woods Institute for the Environment, 99 Pacific Street, Monterey, California, United States of America; 3 Fisheries Ecology Research Lab, Department of Biology, University of Hawai‘i at Mānoa, Honolulu, Hawaii, United States of America; 4 Department of Natural Resources and Environmental Management, University of Hawai‘i at Mānoa, Honolulu, Hawaii, United States of America; 5 The Nature Conservancy of Hawai‘i, 923 Nu‘uanu Avenue, Honolulu, Hawaii, United States of America; 6 Hui Aloha Kīholo, Kīholo Bay, Hawaii, United States of America; 7 Pristine Seas, National Geographic Society, Washington, District of Columbia, United States of America; Aristotle University of Thessaloniki, GREECE

## Abstract

Ocean and coastal ecosystems provide critical fisheries, coastal protection, and cultural benefits to communities worldwide, but these services are diminishing due to local and global threats. In response, place-based strategies involve communities and resource users in management have proliferated. Here, we present a transferable community-based approach to assess the social and ecological factors affecting resource sustainability and food security in a small-scale, coral reef fishery. Our results show that this small-scale fishery provides large-scale benefits to communities, including 7,353 ± 1547 kg yr^-1^ (mean ± SE) of seafood per year, equating to >30,000 meals with an economic value of $78,432. The vast majority of the catch is used for subsistence, contributing to community food security: 58% is kept, 33.5% is given away, and 8.5% is sold. Our spatial analysis assesses the geographic distribution of community beneficiaries from the fishery (the “food shed” for the fishery), and we document that 20% of seafood procured from the fishery is used for sociocultural events that are important for social cohesion. This approach provides a method for assessing social, economic, and cultural values provided by small-scale food systems, as well as important contributions to food security, with significant implications for conservation and management. This interdisciplinary effort aims to demonstrate a transferable participatory research approach useful for resource-dependent communities as they cope with socioeconomic, cultural, and environmental change.

## Introduction

Coral reef fisheries provide critical livelihoods and food that support coastal communities and economies for millions worldwide [1–3]. However, reefs continue to decline due to the combined impacts from pollution, overfishing, climate change, introduction of invasive species, and other stressors [4–6]. These threats undermine the economic, social, and cultural benefits provided by coral reef fisheries, including important food security functions, cultural practices, and livelihoods [7,8].

In the Asia-Pacific region, increased globalization and socioeconomic development is shifting the modes of resource use in traditional fisheries, with implications for human wellbeing, livelihoods, and conservation [9–12]. In reef fisheries, a complex and poorly understood set of social, cultural, and economic factors affect the supply, value, and networks of trade that influence subsistence use, as well as commercial harvest. The processes, markets, and actors involved in seafood value and supply chains are gaining increased attention from scholars and practitioners working in a wide variety of locations [13–16].

The complex dynamics of subsistence, commercial, and cultural use need to be understood, particularly as market-based solutions and supply chain interventions continue to gain momentum in the conservation community. As conservation practitioners increasingly seek to implement such strategies, data limitations can preclude the development of viable solutions intended to increase environmental sustainability and secure social benefits [17–20]. For example, in most coral reefs little is known about the fishing effort and total production [20,21], the supply chain actors and processes that influence markets [14,22], or the diverse values that fisheries provide to local communities and economic markets [23–27]. Further, most tropical countries have limited capacity to monitor, assess, and manage coral reef fisheries [15,28–30].

These gaps in knowledge and capacity impede the development of viable management approaches that seek to promote effective governance and the sustainable flow of benefits from fisheries to resource-dependent communities and markets. As such, there is an immediate need to develop practical approaches to assess both ecological and social dimensions of these fisheries to support culturally appropriate conservation and sustainable development strategies. Here, we present a participatory, community-based method to assess the ecological and social dynamics of a small-scale fishery, using a social-ecological systems approach. Our objectives for this study were to assess the primary factors that influence community food security, including ecological factors (standing stock biomass, habitat distribution) as well as social factors (fishery production modes, distribution of seafood, cultural drivers). We also sought to assess the diverse social, economic, and cultural values associated with this small-scale fishery in order to establish a baseline and targets for future management to sustain these benefits through community-based management. Our overarching goal is to develop a transferable community-based approach that can aid practitioners, researchers, and managers to increase the effectiveness of natural resource management in the face of socioeconomic, cultural, and environmental change.

## Methods

Our research program was developed through a participatory process with community partners. Participatory research describes a suite of approaches that involve researchers, conservation practitioners, and community members working collaboratively in the visioning, goal-setting, design, data-gathering, and assessment phases of research [31]. Such approaches have been shown to yield valid data that are useful for community planning and management [32–34]. In contexts where the research is directed towards a community-based planning effort, such approaches can ensure that research products directly address community needs and inform their planning and management efforts.

**Fig 1:**
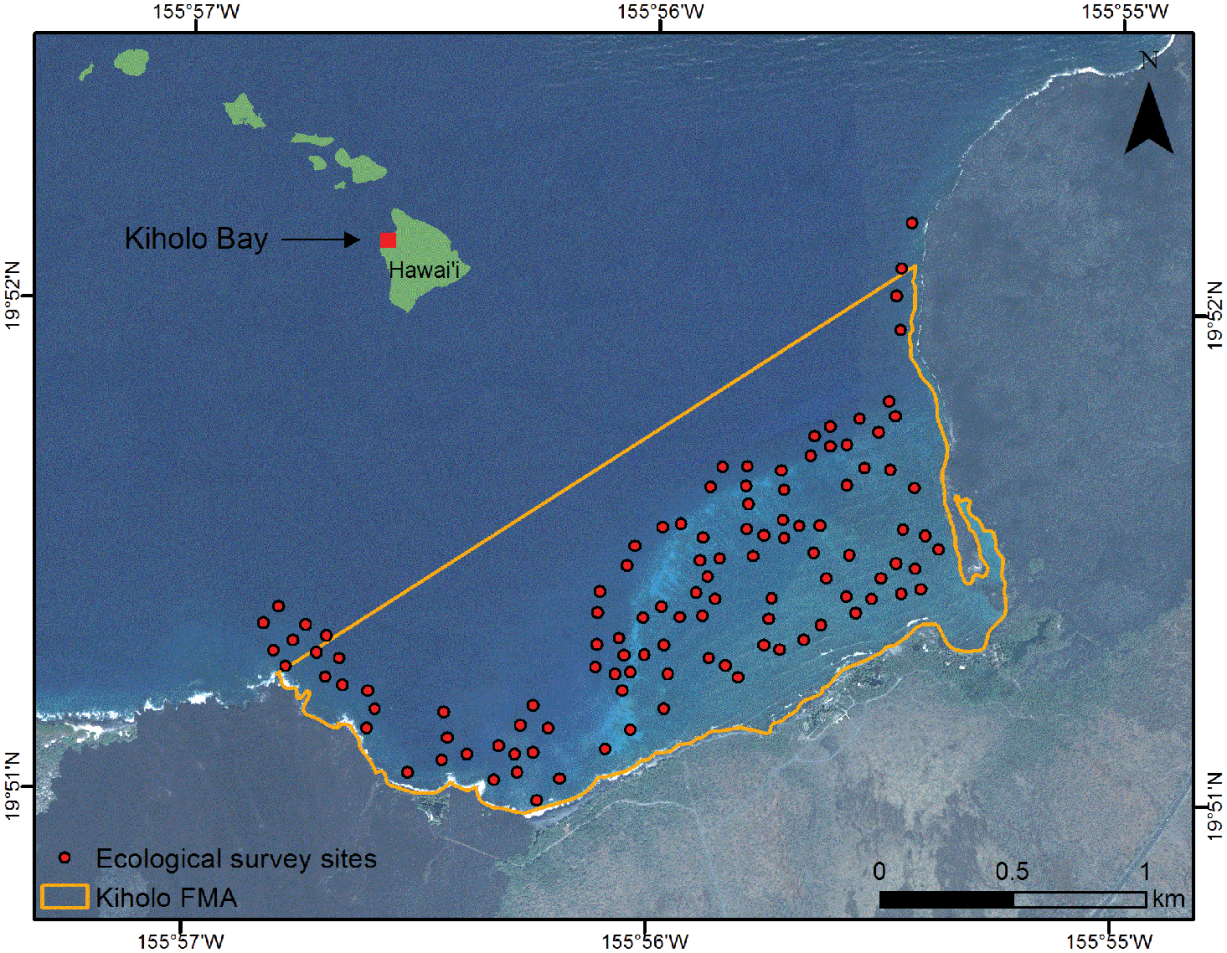
Kīholo Bay study area, including spatial delineation of sampling area for creel and fish flow surveys (orange outline) and locations of transects for ecological surveys of reef fish. Background imagery shows the spatial configuration of the bay and the reef complex, and inset shows the location in the Hawaiian Islands.

This research focuses on Kīholo Bay (19° 51’ 36.41” N, 155° 55’ 59.25” W), a 2.6 km^2^ coastal embayment on the arid leeward side of Hawai‘i Island, with most of the land fronting the bay encompassed in a state park (Fig 1). There is a single access point for vehicles to the state park, as well as a footpath for access from the main road. Kīholo Bay has a rich natural and cultural history, including a well-developed Hawaiian fishpond (*loko i‘a*) complex. The West Hawaii region encompasses the West Hawai‘i Regional Fishery Management Area (WHRFMA), which is managed by the State of Hawai‘i’s Department of Land and Natural Resources (DLNR), Division of Aquatic Resources (DAR). The WHRFMA has specific rules that apply to the entire region, including prohibitions on take of several species including rays and sharks, bans on spearfishing with scuba, restrictions on aquarium species harvest and collection permit requirements, and specific gear restrictions for nets [35]. In addition to these rules, the region also has several marine managed area designations in which specific rules apply. These include Marine Life Conservation Districts (MLCDs), which are marine protected areas that restrict most harvesting activities, Fishing Replenishment Areas (FRAs), which restrict harvesting of most aquarium species, and Fisheries Management Areas (FMAs), which have specific rules that vary by place. Kīholo Bay is designated as a FMA, and in addition to the general rules that apply under the WHRFMA, fish feeding and the use of gill nets are also prohibited [36].

This research presented here derives from a collaborative effort among Hui Aloha Kīholo, a community-based group dedicated to caring for Kīholo Bay, environmental non-profits (Conservation International’s Hawai‘i program and The Nature Conservancy of Hawai‘i), and the University of Hawai‘i at Mānoa. Below, we provide more detail on methods for specific research activities; additional information is available in the supporting online materials (SOM).

### Ecological surveys

Reef fish assemblages were assessed in October 2012 at 112 locations within Kīholo Bay (Fig 1) using standard underwater visual belt transect survey methods [37–39]. Surveys were located on hard bottom habitat and were randomly stratified by benthic habitat (e.g., aggregated reef, pavement with sand channels, rock/boulder [40]), proportional to area present. Two divers swam parallel 25 x 5 m transects at a constant speed and identified to the lowest possible taxon all fishes visible within 2.5 m to either side of the centerline (125-m^2^ transect area). Swimming duration varied from 10–15 min, depending on habitat complexity and fish abundance. Total length (TL) of fishes was estimated to the nearest 5 cm and converted to weight using a length–mass relationship, W = aTL^b^, where the parameters *a* and *b* are constants for the allometric growth equation, TL is total length in cm, and W is mass in grams. Species-specific values for *a* and *b* constants were obtained from fishbase.org and from additional sources from site-based research (A. Friedlander, unpublished data). Paired transect values were averaged at each location.

Ecological surveys allowed us to characterize the reef fish assemblage in a spatial “seascape” approach, using data from in situ transects coupled with benthic habitat maps that delineated habitat structure and cover [40]. This approach allowed us to characterize fish biomass estimates across different habitat types that were surveyed. These results were developed specifically for community use in management, and are considered proprietary information as they contain sensitive information about the species assemblage, habitats, and locations of key fisheries resources in Kīholo Bay.

An estimate of standing stock for fisheries resources, i.e., expanded biomass, was calculated as the average biomass density for each habitat structure category multiplied by the total area of that habitat in Kīholo Bay. We compared the average biomass of resource species (i.e., species targeted in the fishery) at Kīholo Bay with resource fish biomass at several reference sites including: (1) the Northwestern Hawaiian Islands (NWHI)–a remote no-take marine reserve protected as the Papahānaumokuākea Marine National Monument, which is considered an intact and healthy reef ecosystem [41]; (2) Kaho‘olawe—an unfished island in the main Hawaiian Islands protected as part of the Kaho‘olawe Island Reserve (KIR); (3) a set of marine protected areas in the West Hawai‘i region primarily comprised of well-managed Marine Life Conservation Districts (MLCDs), where fishing is restricted [35] (for a complete list of sites included in this category, see S1 Table); and, (4) areas open to fishing in West Hawai‘i (i.e., outside of the Kīholo FMA but subject to general rules and regulations under the WHRFMA). Reference site data for resource fish biomass was calculated using the database of Hawai‘i reef fish surveys compiled by the Fisheries Ecology Research Lab at University of Hawai‘i-Mānoa. We used analysis of variance (ANOVA) followed by Tukey HSD multiple comparison test to determine if differences in biomass between sites were statistically significant.

### Creel and fish flow surveys

We developed a dual component creel and fish flow survey to estimate fishing effort, catch, and post-landings disposition and distribution of seafood (“fish flow”). Fisheries creel surveys (or *‘pakini’*, in Hawaiian) are sampling surveys that target catch, gear type, and fishing effort information from fishers. These data are used to estimate the total catch and effort for that fishery (commercial and non-commercial) and help inform harvest management strategies [42]. Creel surveys are time- and labor-intensive [43,44], but are the most accurate method available for quantitative assessments for data-poor fisheries. Additionally, this method provides opportunities for surveyors to interact with the fishing community on a personal basis, engaging in dialogue with fishers, and providing opportunities to educate the public on management issues [45].

Our creel survey used a temporally and spatially stratified random sample (see S2 and S3 Tables for surveys forms), coupling remote visual observation of fishing activities (location, gear types, frequency) with intercept surveys of fishers to collect data on catch composition, size, and weight, following previously used methods [18,46]. For remote observations, community volunteers conducted dawn-to-dusk surveys for 2–3 weekdays and 2–3 weekend days per month (4–6 total person-days per month) using a high-powered scope and binoculars to record spatial and temporal fishing patterns and gear types used, which together comprise fishing effort.

The observation portion of the survey was paired with an intercept survey. Community surveyors approached fishers exiting the area and conducted intercept surveys to gather information from individual fishers on catch, gear, and fish flow. The fish flow (or *‘mahele’*) survey component targeted information on catch (size, number, total weight), as well as the disposition or end use of seafood, including the geographical location of any recipient(s) of the catch, and disposition categories: 1) kept (for home consumption), 2) given away (for home consumption), 3) sold (or bartered), and 4) released. Additionally, respondents indicated if the catch or some proportion of the catch were to be used specifically for social or cultural events (*pā‘ina*). Our complete creel and fish flow survey instrument are in S2 and S3 Tables.

The observations and intercept surveys were conducted for a 1-year period (1 May 2012–30 April 2013), allowing us to assess the total annual catch and differentiate patterns in fishing intensity and activities [18]. This survey method captures shore-based fishing effort and catch, but records of boat-based activities are limited to effort only (see our section below on caveats to this method).

Catch data were aggregated by trophic group (i.e., apex predators, secondary consumers, herbivores, planktivores, and ‘other’, which included invertebrates). Fish flow survey data were analyzed by disposition category (i.e., kept, given away, sold), weight, number of recorded events, and geographical location. We also performed a spatial analysis to assess the spatial dynamics of seafood distribution in relation to the fishery, by estimating how much of the seafood once caught stayed within a 25-, 50, and 75-km radius from Kīholo Bay.

### Analytical methods

We estimated total fishing effort for Kīholo Bay by quantifying the total daytime fishing hours for each gear type (e.g., spear, thrownet, line) observed in each survey, for each quarter of the year. The total fishing effort was then calculated by multiplying the mean daily fishing effort by the total number of days for each quarter (S4 and S5 Tables). Catch per unit effort (CPUE) for each gear type was calculated by 1) summing (for all days in each quarter) the result of the division of catch for each fisher on a given day by the corresponding fishing effort by that fisher with that gear type on that day, and then 2) dividing by the sum of the number of fishers who were interviewed during that quarter. Using the quarterly CPUE then allowed us to estimate the average catch per day per gear type for each quarter (S6 Table). Total catch was estimated as a product of the total fishing effort and CPUE for each gear type by each quarter (S7 Table). To calculate the total expanded catch for the year, we then summed the catch for each quarter. For error estimation on the total expanded catch, refer to S1 Dataset).

Species level composition of catch for each gear type was calculated by multiplying the species’ proportion in the recorded catch to the corresponding gear’s expanded catch. We report the total expanded catch by fishing method and trophic level.

To estimate the total economic value of the fishery per year, we used the species-level estimate of total reconstructed catch along with species-specific price data collected from local fish markets on the island of O‘ahu. Fisheries markets were surveyed for 38 days (22 weekdays, 16 weekends including the major holidays—Thanksgiving, Christmas and New Year's Day) over 14 months between November 2012 to December 2013 (The Nature Conservancy of Hawai‘i, unpublished data). Other research shows that market prices for reef species an vary slightly from island to island [47], and there was a notable range, spanning more than $10 in per-pound prices for different species. Nonetheless, as these Oahu-based surveys recorded the listed price for every reef fish species available in local markets, this database provided the most accurate information available to approximate the market value of the Kīholo fishery.

To estimate the food provisioning value provided by the fishery, we converted total weight for whole fish to edible weight using species-specific estimates provided by local fishermen (S8 Table). We then used an average portion size of 6 ounces (0.17 kg; or the amount typically served in a restaurant) and the total edible weight of the fishery to derive the total number of meals provided by the fishery. We also estimated the nutritional benefit of fish-derived omega-3 fatty acid consumption via this fishery by using an American Heart Association’s (AHA) recommended 6 ounces of fish per week [48]. All data for figures and tables in this study is publically available at S2 Dataset.

### Caveats of this Method

We developed this participatory method in order to create an adaptable and transferable approach that other conservation practitioners, managers, or resource users could apply to local food systems. We highlight several caveats of this method, which may prove useful both in interpreting our results, as well as adapting this approach to other geographies and contexts. First, we did not include substantial surveying at night, and because our survey design targeted shore-based fisheries, we also were unable to assess catch associated with boat-based fishing activities. In our system, most of the effort in our coral reef and near-shore fishery derived from shore-based activities, but in adapting this approach to other geographies the design and sampling strategy may need to be adjusted accordingly. Also, our survey probably underestimates commercial activities, because it relies on fishers voluntarily reporting and the intercept survey method may create social pressure to not report all sold catch as this can be perceived as less culturally acceptable than subsistence-oriented fishing.

Second, our method requires significant effort by community surveyors (multiple patrol days per month, over a 1-year period) as well as for the aggregation, quality control, and analysis of fishery data. Community surveyors dedicated significant time in the initiation of the project to socialize the project concept, intent, and design with the fishing community, and remained diligent in surveying activities, which was key to the project success. Similarly, a dedicated interdisciplinary team worked hand-in-hand with community members to analyze the data at scales that were meaningful for the fishing community, and established protocols to safeguard the dataset, which remains the intellectual property of the community organization. In order to reduce the effort required for data collection, quality control, and analysis, we are piloting a mobile data portal and waterproof digital tablet for our survey forms (CI Hawai‘i’s #TEK+TECH project), which if successful will allow this approach to be less resource-intensive and more scalable.

## Results

### Ecological profile of Kīholo Bay

Kīholo Bay benthic habitats consists of rock/boulder (1.15 km^2^, 45%), followed by pavement with sand channels (0.44 km^2^, 17%), aggregate reef (0.25 km^2^, 10%), and sand (0.71 km^2^, 28%) (Table 1). Rock/boulder had the highest expanded biomass due to its larger area (Table 1). Total standing stock of resource fishes in Kīholo Bay, calculated using benthic structure, was 48,337 kg. This is a highly conservative estimate since it does not take into account unsurveyed sandy bottom habitat. The reef fish community in Kiholo is comprised of secondary consumers (48.9%), followed by herbivores (38.6%), and planktivores (9.7%), with apex predators accounting for only 2.7% of the total.

**Table 1:** Area, number of survey sites, and average biomass for each habitat structure category surveyed in Kīholo Bay. Sand habitat was not surveyed and not included in this table, though it makes up an additional 0.71 km^2^ of habitat area (28%) in the bay. SE is standard error of the mean for transect biomass values in each category. Average biomass was expanded to the total area of each habitat structure category by trophic group, resulting in an estimate of total standing stock of resource fishes.

					*Expanded Total Biomass by Trophic Group (kg)*				
*Structure*	*Area (km* ^*2*^ *)*	*# Survey Sites*	*Average biomass (g m* ^*-2*^ *)*	*±SE*	*Apex Predators*	*Herbivores*	*Secondary Consumers*	*Planktivores*	*Total*
Rock/Boulder	1.15	58	25.3	2.8	1,418	13,310	11,194	3,180	29,103
Pavement with Sand Channels	0.44	36	22.9	3.6	0	3,611	5,525	979	10,115
Aggregate Reef	0.25	18	36.3	5.3	120	2,405	5,944	650	9,118
Sand	0.71	N/A	N/A	N/A	N/A	N/A	N/A	N/A	**N/A**
Total	2.55	112			1,538	19,326	22,663	4,810	**48,337**

**Fig 2:**
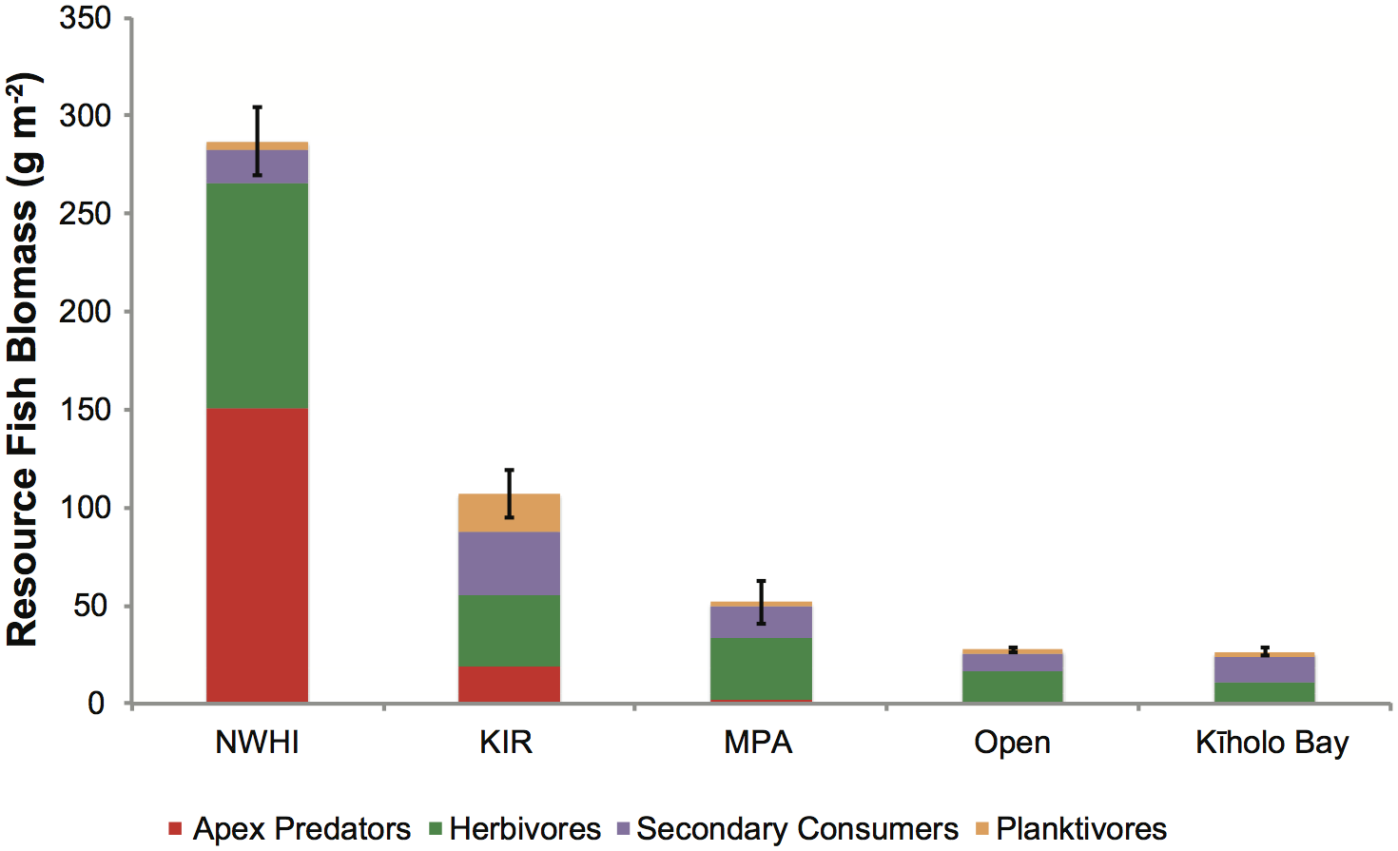
Average biomass density by trophic group for resource fishes in the Northwestern Hawaiian Islands (NWHI), Kaho‘olawe Island Reserve (KIR, an unfished reserve in the main Hawaiian Islands), marine protected areas (MPA) in the West Hawaii region, areas open to fishing in West Hawai‘i (referred to as ‘open’), and in Kīholo Bay. MPAs included in this comparison are included in S1 Table. The multiple comparisons test suggest that resource fish biomass at Kīholo Bay was not significantly different from that of highly managed areas in West Hawai‘i or the sites which have little or no fishing regulations, which we refer to as ‘open’ (p = 0.07).

The *average* biomass of resource fishes for all of Kīholo Bay was 26.3 g m^-2^ (Fig 2), which is significantly lower than marine reserve reference sites in the Northwestern Hawaiian Islands (287 g m^-2^) and Kaho‘olawe (107 g m^-2^) (Fig 2). Average biomass in Kīholo Bay was about half of that found in highly managed areas (i.e., the average of resource biomass across the following sites within in WHRFMA: Old Kona Airport, Kealakekua Bay, Lapakahi, and Waialea Bay) (51.7 g m^-2^) but similar to areas open to fishing in West Hawai‘i region (outside of the Kīholo FMA and the mentioned managed areas) (27.6 g m^-2^). The multiple comparisons test suggest that resource fish biomass at Kīholo Bay was not significantly different from that of highly managed areas in West Hawai‘i or the sites which have little or no fishing regulations, which we refer to as ‘open’ (p = 0.07, Fig 2).

### Linking ecology, catch, and fishing method

We estimated the total annual expanded daytime catch from Kīholo Bay to be 7,353 ± 1547 kg yr^-1^ (mean ± SE), or 15.2% of the estimated standing stock biomass of resource fishes of Kīholo Bay (48,337 kg). We documented each gear and method used, and calculated the total effort and catch per unit effort (CPUE) by gear type (Table 2). A total of 29 different species were harvested and were grouped by trophic category.

**Table 2:** Dominant fishing methods, total effort (fishing hours), and catch per unit effort (CPUE in kg day^-1^) with means and coefficient of variation (COV) from the one-year creel survey at Kīholo Bay. Number of observed events is also included. Line fishing includes hand pole, rod-and-reel, and flyfishing. Spear includes 3-prong pole spear and band-powered speargun. Other methods include limpet (*‘opihi*, in Hawaiian) gleaning, crabbing, aquarium fishing.

*Fishing method*	*Gear Type*	*Fishing Hours*	*CPUE*		*N*
			*Mean*	*COV*	
Line Fishing	Hand pole	2745	7.5	0.9	21
Line Fishing	Rod and reel	2259	6.2	0.1	17
Thrownet	Thrownet	1434	3.9	0.09	43
Spear	3-prong; speargun	799	2.2	0.05	15
Other	Limpet (Opihi) Gleaning	460	1.3	0.06	6
Other	Crabbing	146	0.4	0.02	3
Other	Aquarium	84	0.2	0.01	1
Other	Other	88	0.4	0.02	1

**Fig 3:**
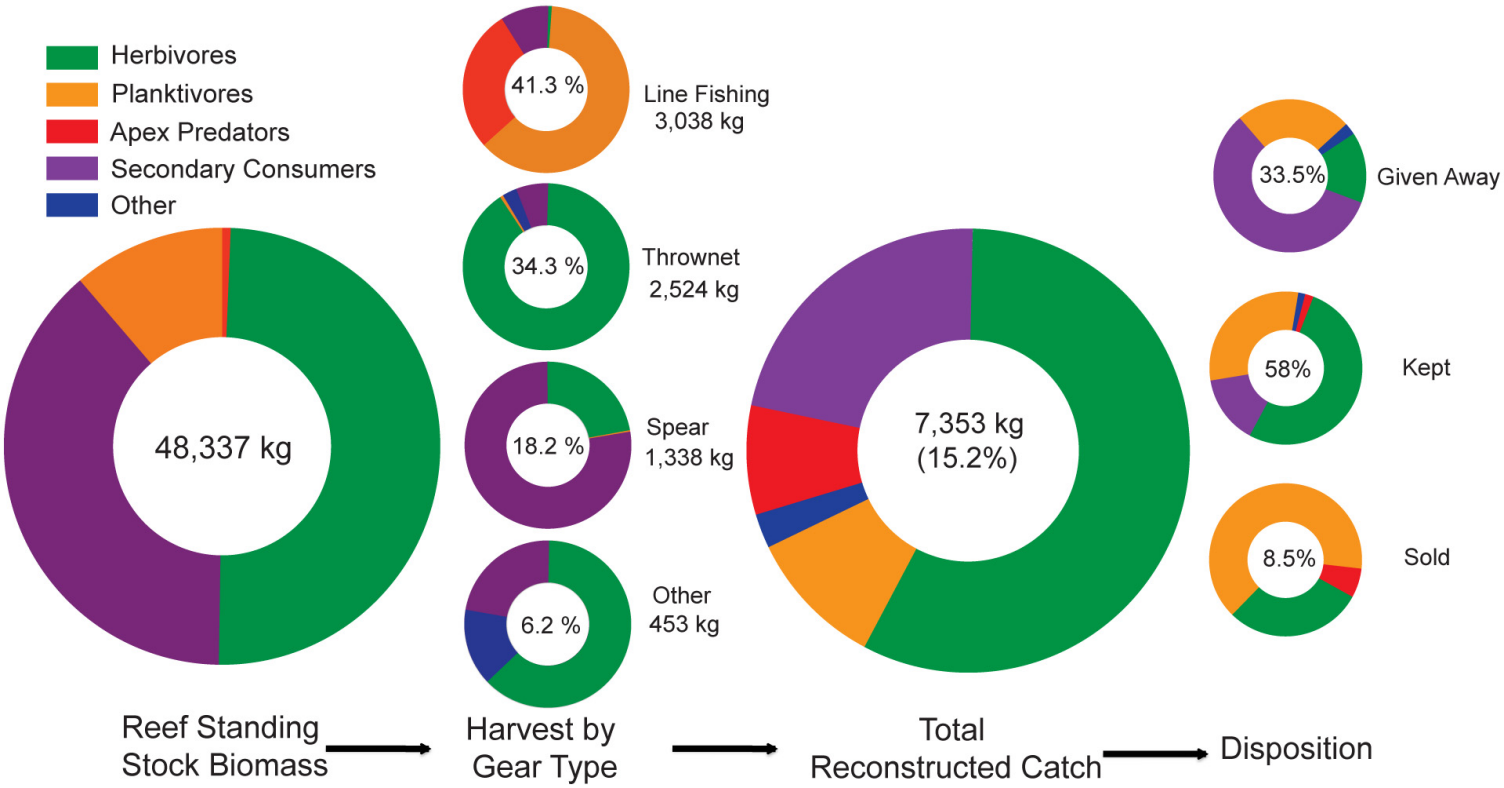
Fish flow from reef to table for Kīholo Bay, with variation in composition of key trophic groups throughout these artisanal supply chains. The first pie chart shows the total biomass, by tropic group, of reef fish in Kīholo Bay, as determined from in-water ecological surveys. Next, the harvest by gear type is depicted, showing how different gear types target different mixes of trophic groups; the total % of total harvest by each gear type is included in the center of each pie chart. The total expanded catch is approximately 15.2% of the standing stock biomass, and the proportions of the catch vary in comparison to the standing stock trophic composition. Finally, the last three pie charts show which trophic groups are distributed to which end use (disposition); percentages indicate the proportion of the total catch directed toward each end use (given away, kept, sold).

Gear types used at Kīholo Bay (Table 2) were combined into four broad categories (i.e., line fishing, thrownet [castnet], spear, and other) to summarize the annual reconstructed catch by gear type and trophic level (Table 3). Apex predators were rare in our ecological surveys (<1% of total biomass), but accounted for 11.5% of harvested species by weight (Fig 3). Thrownet and spear fishing had the highest CPUEs (Table 2), and accounted for 50% of the total expanded catch (Table 3), with two-thirds of the combined thrownet and spear fishing catch were herbivores (Table 3, Fig 3). Line fishing caught 41% of the expanded catch, mostly planktivores (62% of line fishing catch), followed by apex predators (28% of line fishing catch). Thrownets accounted for 34% of the total catch, and almost all the thrownet catch were herbivores (90%). Secondary consumers represented 49% of the estimated standing stock and comprised 22% of catch landed. Most of the secondary consumers were taken by spearing (66% of the expanded secondary consumer catch, and 14% of the total expanded catch), followed by thrownets (10% of the expanded secondary consumer catch) (Fig 3). Invertebrates (e.g., limpets [*‘opihi*], crab, etc.) accounted for 5% of the total expanded catch and were caught by gleaning.

**Table 3:** Total reconstructed catch (in kilograms) for May 1, 2012 –April 30, 2013 for Kīholo Bay by gear type and trophic group.

*This study*, *Kīholo Bay*, *2012–2013*	*Apex predators*	*Secondary Consumer*	*Herbivore*	*Planktivore*	*Other (including invertebrates)*	*Total by gear type*	*% of total by gear type*
Line Fishing	843	284	20	1891	0	3038	41.3
Thrownet	0	159	2279	16	70	2524	34.3
Spear	0	1038	297	3	0	1338	18.2
Other	0	102	284	0	67	453	6.2
**Total by trophic group**	**843**	**1583**	**2880**	**1909**	**137**	***7353***	
**% of total by trophic group**	**11**	**22**	**39**	**26**	**2**		

**Fig 4:**
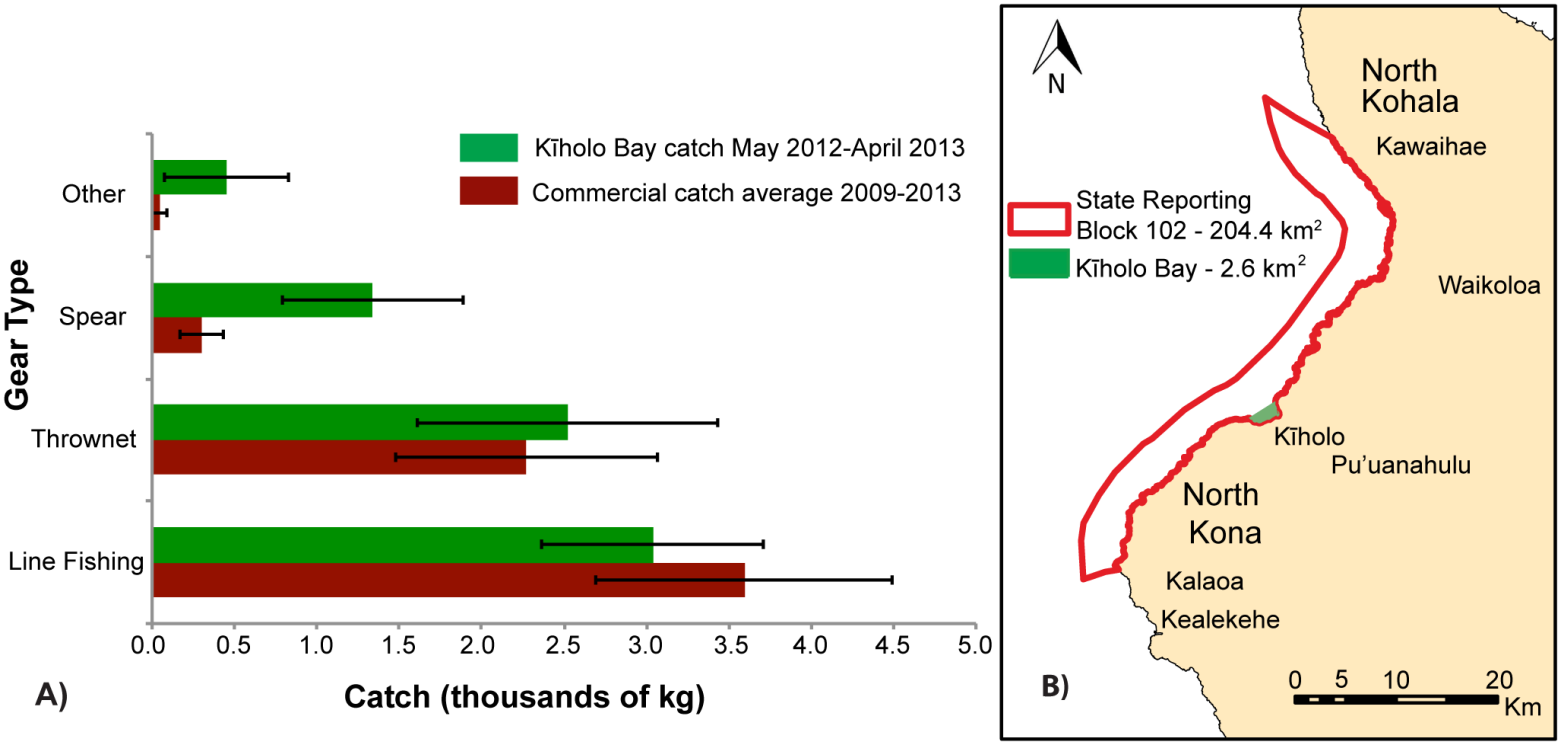
Coastal fisheries catch from creel surveying efforts in Kīholo Bay and the State of Hawai‘i commercial reporting block. (A) Total reconstructed annual catch (in kg) by gear type from a one-year creel survey at Kīholo Bay, Hawai‘i (red) compared to a 5-year annual mean of commercial marine landings by gear type (2009–2013, red) reported to the State of Hawai‘i’s Department of Land and Natural Resources for the entirety of reporting area 102. (B) Size of Kīholo Bay in reference to the DLNR reporting area 102 for commercial catches. The DAR reporting block is approximately 78 times larger than the reporting area for the Kīholo creel survey. Although the commercial catch and the Kīholo Bay catch represent very different spatial scales, the total annual catches are not significantly different (t-test: t = 1.5934, DF = 4, p = 0.1863). The category “other” includes gleaning for Kīholo Bay and trolling for commercial data. Line fishing includes handpole and rod-and-reel (which includes whipping, dunking and slide-baiting). Gleaning includes invertebrate collection.

Total expanded catch from Kīholo Bay (7,353 ± 1547 kg yr^-1^), comes from an area of ca. 2.6 km^2^. This area is roughly 78 times smaller than the corresponding State of Hawai‘i commercial fishing reporting area (204 km^2^) for this region. However, the catch from Kīholo Bay is 17% higher than the 5-year average (6,218 ± 712 kg yr^-1^, 2009–13) of commercial catch reported to Hawai‘i’s Department of Land and Natural Resources’ (DLNR) Division of Aquatic Resources (DAR) for the entire 204 km^2^ commercial fishing reporting block (Table 4; S9 and S10 Tables) (Fig 4A, S10 Table). This flags that total catches (commercial and non-commercial) in coastal fisheries in the region are significantly unestimated if only traditional data reporting is considered. The reported commercial catch consists primarily of coastal pelagics (e.g., *‘ōpelu*, mackerel scad, *Decapterus* spp., and *akule*, big-eye scad, *Selar crumenophthalmus*), thus there is very little overlap between species composition of the reported commercial catch and the generally non-commercial catch obtained from our survey (Fig 4B). Table 4 summarizes the reported commercial catch in the region for 2009–2013, and singles out the *‘opelu* in fraction of total volume of catch as well as total market value. Although the commercial catch and the Kīholo Bay catch represent very different spatial scales, the total annual catches are not significantly different (t-test: t = 1.5934, DF = 4, p = 0.1863). Line fishing represents the highest percentage of the reported commercial catch (58% and 41%, respectively). Only 5% of the reported commercial catch came from spearing and no commercial catch was reported from gleaning. This differs from the catch in the Kīholo creel survey, in which spearing accounted for 18% and gleaning for 6% of the total. Nets in the reported commercial catch and the Kīholo catch accounted for 37% and 34% of the catch, respectively; there are no distinctions made between net types used commercially, while the Kīholo creel survey captures thrownets specifically.

**Table 4:** Commercial catch (kg) reported to DAR 2009–2013 for the commercial reporting block surrounding Kīholo, with mean and standard error (SE). Total value was computed based on species-level market prices obtained by The Nature Conservancy through market surveys (unpubl.). ‘Opelu (mackerel scad, *Decapterus* spp) fraction of volume and value is also listed.

*Year*	*Total catch (kg)*	*Total value ($)*	‘ōpelu (*Decapterus* spp) *fraction of catch (%)*	‘ōpelu (*Decapterus* spp) *fraction of value (%)*
2009	4,246	48,483	51	44
2010	6,859	78,794	56	49
2011	8,380	100,538	48	40
2012	6,448	75,992	62	53
2013	5,159	54,054	71	68
Mean	6,218	71,572	58	51
± SE	± 712	± 4,184	± 2	± 2

### Fish flow and seafood supply chains

We focus our analysis (Table 5) on the 76% of total catch data where the creel survey recorded information on disposition (end use of the seafood). The remaining catch did not have disposition information (24%) and consisted predominantly of herbivores (44%) and secondary consumers (43%). It is not certain why disposition information was not provided in some cases, but it is possible that fishers from outside of the community of Kīholo may have withheld this information because they did not want to disclose it to the surveyor.

**Table 5:** Weight of total reported catch and reported catch with post-landings disposition information, by trophic group, from Kīholo Bay surveys.

*Total Reported Catch (kg)*			*Total Catch with Reported End-use Information (kg)*				
*Trophic Group*	*Weight (kg)*	*Fraction of total reported Catch (%)*	*Weight (kg)*	*Fraction of total reported catch that contain end use data (%)*	*Given Away (kg)*	*Kept (kg)*	*Sold (kg)*
Apex Predators	21.1	4.0	6.6	1.6	0	1.1	0.5
Secondary Consumers	415.3	35.6	114.6	33.5	19.4	8.4	0
Herbivores	404.7	34.7	155.1	32.0	5.1	30.1	2.5
Planktivores	284.7	24.4	128.5	31.2	8.2	17.5	5.5
Other	15.1	1.3	6.8	1.7	0.8	0.8	0
			**Breakdown by end-use:**		33.5%	58%	8.5%

**Fig 5:**
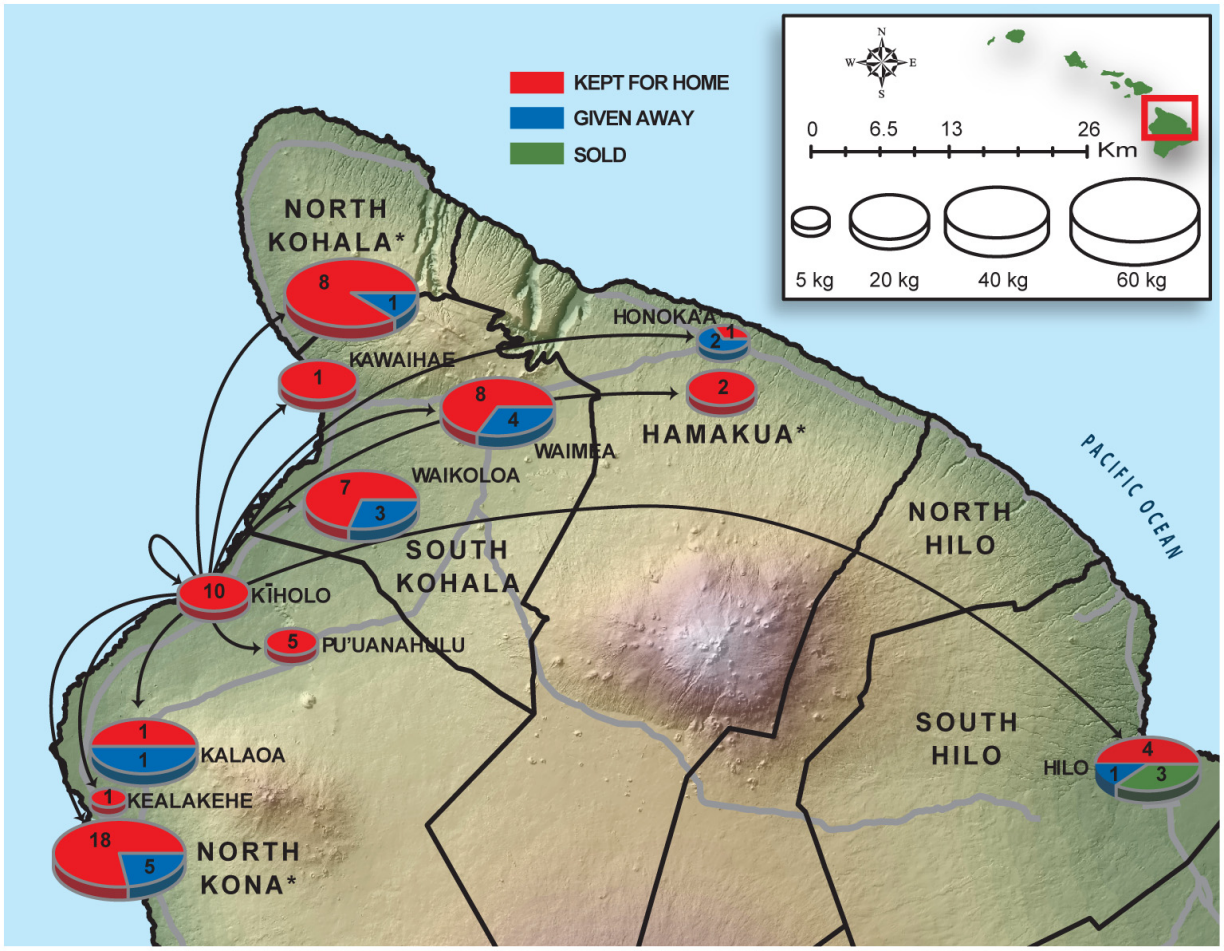
Mapping artisanal seafood supply chains as “fish flow” from the Kīholo Bay coral reef fishery. Arrows indicate locations where seafood from Kīholo is consumed. Exact locations are indicated as place-names; some destinations were only available at coarser district (moku) levels (these are indicated with asterisks). Post-landings disposition is distinguished for kept (red), given away (blue), and sold (green) seafood. Pie charts are scaled to the total catch (kg) for each destination. The numbers in each pie are the number of distribution events recorded for each destination and represent only survey-recorded end-use, not the annual expanded catch. The district boundaries, Digital Elevation Model (DEM), and transportation lines were acquired from the Hawai‘i state GIS portal [http://planning.hawaii.gov/gis/download-gis-data/].

Using data from our fish flow surveys, we mapped the geographic location, amount (by weight and number of recorded events), and disposition (end use) for seafood obtained from the Kīholo Bay coral reef fishery (Fig 5). This “fish flow” mapping revealed the geographic scale and dynamics of artisanal seafood supply chains associated with this fishery. Most seafood is consumed relatively close to Kīholo Bay, with 92% percent of the catch remaining within a 75-km radius of Kīholo Bay. Within this 75-km radius, 33% of the catch is given away, 53% is kept, and 6% is sold.

Overall, the amount of catch that was reported as kept is nearly twice the amount given away. Subsistence/consumptive uses (kept + given away) accounted for the vast majority of seafood use in this fishery (91.4%), with the remainder going to commercial markets (Table 5). Trophic group composition of the catch differed based on disposition. Secondary consumers and planktivores dominated the catch that were given away, herbivores dominate the catch that was kept for home consumption, and planktivores and herbivores were the primary species sold in commercial markets (Fig 3).

### Fishery Valuation

#### Market-equivalent economic value of the Kīholo fishery

Using the total catch by species and species-specific market price data, we calculated the total annual market-equivalent value of the fishery in Kīholo to be $78,432. This includes the total expanded catch across all disposition (end use) categories. We note that a very small fraction of the Kīholo Bay fishery actually enters the formal cash economy as commercially sold seafood (~8.5%). More than a third of the total market-equivalent value is comprised of herbivores ($27,059) (Table 6), secondary consumers contributed 29.8% to the total value ($23,373), planktivores 21.0% ($16,470), and apex predators 14.7% ($11,530). In comparison, the average total annual economic value of the commercial catch reported to DLNR for the entire commercial reporting block over the period 2009–2013 was $71,572 ± 20,920 SD (Table 4, S7 Table). Both in terms of weight and economic value, the commercial catch was largely dominated by *‘ōpelu* (mackerel scad *Decapterus* spp.), which was not a target fishery in Kīholo Bay during our survey year (Table 4). The total annual market-equivalent value of the Kīholo Bay fishery rivals that of the commercial fishery, which occupies a reporting block area more than 75 times larger than Kīholo Bay.

**Table 6:** Economic and nutritional value of total reconstructed catch for Kīholo Bay (May 1, 2012—April 30, 2013). MP is market price. Expanded catch by species was paired with market value (TNC, unpublished data). Number of meals assumes 6-ounce servings. Fraction of meals is essentially the percentage of meals delivered by each trophic group for this study.

*Trophic Group*	*Economic Value based on MP*	*Fraction of total economic value (%)*	*Nutritional value (no*. *meals)*	*Fraction of meals (%)*
Apex Predators	$ 12,447	16%	3,321	11%
Secondary Consumers	$ 18,889	24%	6,114	20%
Herbivores	$ 29,247	37%	10,945	36%
Planktivores	$ 17,849	23%	10,107	33%
TOTAL	$ 78,432	100%	30,487	100%

#### Food provisioning value of the fishery

By converting the total fish weight to edible weight (S8 Table) and using a standard portion size of 6 oz (0.17 kg), we estimated the total number of meals provided by this fishery at 30,487 per year (Table 6). Herbivores comprised 36% of the meals, followed by planktivores (33%), secondary consumers (20%), and apex predators (11%). We also assessed a key metric of the nutritional value of the fishery—the omega-3 fatty acids provided by seafood harvested in this fishery. Assuming that species harvested have a similar amount of omega-3 fatty acids per pound, we estimate that the Kīholo fishery provides enough omega-3 fatty acids to support the daily requirements for 586 people over a year, based on guidelines from the American Heart Association [48]. This demonstrates that the Kīholo fishery has significant nutritional value and may play an important role in sustainable foods and diets in this region.

#### Cultural values of the fishery

To obtain indicators of the cultural value of the fishery, we assessed the prevalence of two key practices: (1) The fraction of catch that fishers indicated was to be used for social gatherings (*pā‘ina*); and, (2) The giving of seafood away to friends, extended family, and community members. Fishers often provide seafood for community cultural events such as birthdays, weddings, or funerals [49]. Additionally, giving seafood to friends and family is part of cultural practice in Hawai‘i and the Pacific Islands region, and is an indicator of the cultural value of the fishery [50]. In total, 21.4% of the catch, which equates to ~6,500 meals yr^-1^, was reported as used for cultural functions (pā‘ina). Additionally, 33% of the total catch is given away, comprising up to ~2,150 kg of seafood annually from Kīholo Bay, or ~10,000 meals.

## Discussion

### Food security, social networks, and artisanal supply chains

We documented the important food provisioning functions associated with this small-scale coral reef fishery, where over 90% of the catch is directed toward household consumption (Fig 4, Fig 5). While much research has been directed toward the role of fisheries in food security [51–53], the food provisioning functions of small-scale fisheries have remained poorly quantified, particularly at local scales [3,54,55]. Studies rarely document food provisioning over significant spatial or temporal scales with accurate, site-based data. They also rarely engage local communities in a truly participatory research approach.

By mapping the distribution of the catch and estimating the magnitude of the benefits provided by this fishery, our research uncovered three important characteristics of the food provisioning function provided by this resource system. First, our map shows the geographic location of resource users and households that benefit from the fishery. In essence, this approach shows how the reef feeds the community—a benefit that is often paramount for resource-dependent communities.

Second, this approach reveals the social network for this small-scale fishery. Resource users for fisheries in pre-contact Hawaii (< 1778 AD) were located in the same watershed or district as the resource base [56], but through a long history of land dispossession, development, and other factors [57], the network of resource users that access this fishery and others like it has become more broadly distributed. This history of displacement and diaspora can make it difficult for resource managers to define the extent of the community of resource users that interact with the target ecosystem—this research illuminates these spatial patterns of social relationships.

Third, this approach reveals the different supply chains that exist in this artisanal fishery, illuminating various supply chain ‘sectors,’ including direct reliance (kept), cultural exchange (given away), and commercial sale market sectors (sold). Understanding these supply chains is important because they reveal that an interacting set of social, cultural, and economic factors that affect the distribution of seafood from reef to table in a small-scale fishery. For example, fishers are economically reliant on the fishery to subsidize their household food budgets directly with harvested catch. This fishery provided ~30,500 meals, with a market value of $78,432 annually (for 7,353 kg of seafood catch), which offsets food budgets for community members. The large seafood harvest that is given away or caught specifically for cultural events also reveals the importance of sociocultural values in motivating fishing activities, a common occurrence in the Asia-Pacific region [49,50,58]. The mixed mode of reliance (subsistence, livelihood, cultural) has been observed in other similar systems with communities highly dependent on local natural resources for food security [13,16,59]. There are several studies that have shown that fishing can carry multiple end-uses, including both food, income, and livelihoods, and can be driven by multiple motivations beyond economic drivers, particularly in locations where historical traditions and socioeconomic factors such as cultural attachment and heritage affect fishing activities [60–62]. Conservation practitioners are increasingly working together with local leaders and resource users to assess the benefits and manage for the long-term food provisioning and cultural functions associated with these fisheries [63–65].

Similar to studies on agro-ecosystems [66–68], our research uncovers the geography of the “food shed” for small-scale fisheries. These findings carry important implications for emerging research in small-scale fisheries focused on the role of markets in structuring human-environmental relationships in these systems. As researchers begin to disentangle the complexity of different market sectors and supply chains for small-scale fisheries, there is a pressing need to identify the interventions that could improve the sustainability of these supply chains and the value they bring to communities, businesses, and environmental sustainability efforts. For example, conservation practitioners are already developing a variety of market-based approaches to create incentives for sustainable harvesting, including eco-labeling and certification schemes, fisheries improvement projects, and other approaches [69–72]. Over the past decade, these types of supply chain interventions have largely been directed toward large-scale industrial fisheries [73–75], but there is significant interest in adapting these approaches to small-scale fishery systems. However, small-scale resource systems carry different environmental, social, and economic attributes, and elucidating how small-scale, artisanal supply chains operate will be critical in developing market-based approaches for small-scale, data-poor fisheries and other similar resource systems. The integration of community-based and market-based approaches, together with local food movements, holds much promise for transitioning small-scale fisheries to sustainability.

### Assessing the ecological sustainability of harvesting

Defining the sustainable limits for resource use is central to any management or conservation plan, but can often be difficult to assess, particularly in small-scale fisheries, where multiple gear types are often used to harvest a multi-stock assemblage of target species [76]. Much recent attention focuses on the use of thresholds for coral reef fisheries management, based on literature that shows most critical ecological functions in coral reefs are preserved if harvesting stays within an envelope of 0.25–0.5 of virgin stock biomass (B_0_) [77,78]. Key to this method is the selection of sites that can serve as reasonable proxies for virgin stock biomass; typically these sites are older, and preferably larger, marine reserves. Selection of these reference sites is critical because they in turn determine the putative sustainability of comparison sites, as determined through thresholds. If these estimates of upper and lower bounds are accurate, this method holds much promise as a management tool by providing clear targets for either stock recovery or maintenance, as well as early warning signs for management.

We compared the average biomass of resource fishes at Kīholo Bay with other sites around the Hawaiian Islands (Fig 2). The archipelago has two potential reference sites, which vary greatly in terms of their biomass: the Northwestern Hawaiian Islands (managed as the Papahānaumokuākea Marine National Monument), and Kaho‘olawe, an island that was off-limits to fishing for more than 50 years when it was used as a target range by the US military and is now managed by the Kaho‘olawe Island Reserve Commission, which restricts almost all fishing activity on reefs. Even though both sites are essentially unfished, Kaho‘olawe and Papahānaumokuākea differ substantially in terms of average biomass, owing primarily to the much higher abundances of apex predators and herbivores in the Northwestern Hawaiian Islands (Fig 2).

As a result of these differences, the interpretation of a threshold-based assessment of the sustainability of Kīholo’s fishery varies significantly depending on the reference site chosen. Kīholo Bay’s average biomass for resource fishes is estimated at 26.3 g m^-2^ and represents 9.2% of the Papahānaumokuākea resource fish biomass, i.e., it is below 0.1xB_0_ (where B_0_ is the reference biomass), and well below the 0.25xB_0_ threshold of sustainability as defined by McClanahan et al. [77]. If Kaho‘olawe is used as a reference site, Kīholo’s biomass is 24.6% of the reference biomass, i.e., roughly equivalent to the 0.25xB_0_ threshold, which indicates that the state of the fishery is at the lower limits for sustainability and that local action is needed to improve the fishery.

These biomass reference comparisons also illustrate a key dilemma for conservation—are appropriate reference sites for conservation those sites that are more pristine or ones that are more realistic (i.e., achievable) [79]? Papahānaumokuākea and remote reefs are often used as an ecological baseline for “pristine” reefs [80,81], and fish community structure in these systems are dominated by a high proportion of apex predators [41]. However, it is unlikely that any marine managed area in the main Hawaiian Islands can achieve these levels of predator biomass, particularly since these areas are under continual human use and have a long historical legacy of human impacts [82,83]. Kaho‘olawe may be a more realistic reference for Kīholo because it has a similar assemblage structure in terms of relative proportion of trophic groups (Fig 2) and represents an achievable level of biomass for a well-managed site that exists in a wider seascape where fishing is prevalent. However, regardless of which site is used as a reference, this analysis indicates that the resource fish biomass of Kīholo Bay is well below key thresholds for sustainability. Like many sites across Hawai‘i and the Pacific region, resource fish biomass and other ecological functions could be compromised if management is ineffective.

Understanding changes in harvesting pressure and biomass over longer time scales will help determine if the standing stock is stable with current levels of use, or if additional management actions are needed to ensure long-term sustainability, particularly given longer-term socioeconomic, demographic, and policy changes in the region. In surveys at various sites around Hawai‘i, fishers often comment on a long-term decline in catch abundance (e.g., [84]). Community elders in Kīholo have suggested that the declines they have seen in marine resources in the bay may be related to increased fishing effort due to the increased shoreline access in recent years (The Nature Conservancy, unpublished data) [56]. There is increased focus on fishery co-management in Hawai‘i and the Asia-Pacific region, and while interest is high, there are still only a few sites statewide where these approaches have been successfully implemented [85,86]. Despite this implementation gap, there is much promise in collaborative management agreements for coral reef fisheries in coastal communities across the globe [87–89]. In sites like Hawai‘i, where place-based management is more congruent with traditional management, these approaches achieve a higher level of social legitimacy. Recent research suggests that co-managed sites are more likely to sustain positive social and ecological benefits if the governance systems take into account key enabling conditions, costs, timelines, community building, and process legitimacy within the planning and implementation effort [85,90,91]. Use of participatory research approaches like the one described in this paper can help communities, researchers, and managers develop successful co-management arrangements.

### Diverse fisheries carry diverse values

Our research highlights a diversity of values associated with this small-scale fishery. First, the fishery carries a significant food provisioning value, providing ~30,500 meals per year to households across the region and beyond (Fig 5), representing an important source of food and nutrition, including omega-3 fatty acids. These meals offset household food budgets for people in the community of resource users and their social networks, with the vast majority of the $78,432 in total annual economic value of the Kīholo fishery directed toward subsistence and consumptive uses. This may be an important function for community members, particularly those in economically disadvantaged households in Hawaii, as it has been shown in other reef-dependent regions [13,14]. Hawai‘i County has a median annual household income of about $65,000, and 9.5% of people live in households below the federal poverty line [92]. The USDA estimates that a family of four requires a monthly food budget of at least $1,088 [93]. Hypothetically, the contribution from this small-scale fishery could offset the food budget costs by 10% for 60 households [94]. Income data were not available for the fishers who participated in the Kīholo Bay fishery, precluding deeper insight into the potential effect of income inequality on fishing effort for consumptive or commercial purposes. More research is needed on the nexus of poverty, governance, food commodity markets, and resource dependence in the Asia-Pacific region, particularly as environmental changes and globalization continue to drive shifts in patterns of subsistence need and market forces that affect livelihoods [15,95,96].

Beyond market-equivalent economic value, this small-scale fishery also carries important social and cultural values for the community. Harvesting and gathering food remains a central aspect of lifestyles in Hawai‘i, driven by a longstanding historical connection to place and practice among Hawaiian communities [97,98]. This fishery, like other resource systems in Hawai‘i and elsewhere, provides a diverse set of cultural ecosystem services for communities, which often are difficult to disentangle from other services (such as food provisioning) and difficult or inappropriate to quantify. We report two indicators of cultural values associated with this fishery, including the fish that are shared or given away as part of cultural practice and the fish that are directly used for social events or functions (*pā‘ina*, literally “meal” or “dinner”). Overall, more than 2,150 kg of seafood were reported as given away, comprising 33.5% of the catch. Approximately 6,500 meals over the course of a year were sourced directly for cultural functions, pointing to important motivations or what is sometimes referred to as “triggers” for fishing events [49]. These community events play a key role in social cohesion, and the provisioning of food for these events draws on important relationships that maintain social and kinship ties among community members and families [49,50,99]. While these indicators only reveal a limited set of the cultural values associated with this fishery, they do suggest that a substantial proportion of fishing events and catch use are associated with cultural practices, providing quantitative metrics for relationships between this resource system and community wellbeing. Our results illustrate the important connections between land and natural resources (*‘āina*), community, and family (*‘ohana*) [100,101] which operate through distribution networks that are influenced by social kinship ties and networks. These relationships have deep historical roots in sociocultural institutions and practices, common not only to Hawai‘i but to the wider Asia-Pacific region as well as other coastal areas globally.

### Implications for management and conservation

There are decades of literature pointing to the benefits of participatory assessment and monitoring, including building cross-sectoral knowledge, engaging community members in the co-production of knowledge, and developing baselines for community planning and management [65,87,102–105]. Beyond these benefits, we uncovered a set of ancillary benefits to this effort that are less commonly reported but have had positive benefits for the community. These include opportunities for outreach directly with fishers as a result of their participation and engagement in monitoring efforts. Participatory research approaches that put community concerns at the forefront of the research design, implementation, and assessment have much greater potential to inform community actions. In the case of Kīholo, the community’s principal objective was to understand how the fishery provides for the fishers and families associated with this site, and to more deeply understand fishing activities as they relate to community-based collective action to steward the resource. Through the survey, community members built relationships with resource users that accessed the area, and as a result were able to learn who was fishing and why, and where they were from, providing important information on the diverse community of stakeholders that interact with the fishery. By engaging with the fishing community to create baselines and datasets on these key community priorities, resource users have begun to change their attitudes and perceptions, and the community is engaging fishers to build trust and the foundation for collective action. Developing an understanding of the pool of resource users and their motivations is a critical precursor to organizing users to engage in collective action for effective management of the resource. These types of “step zero” processes are an understudied aspect of community-based management, as they often help shape the path forward for future engagements and actions [62,106,107]. Further, these place-based approaches are ultimately necessary to support management at geographically relevant scales to ensure the perpetuation of key ecosystem services and benefits that support community well-being [108,109].

Second, we have anecdotal evidence that the presence of community surveyors served to decrease instances of overharvesting and potentially illegal fishing. Notes taken on the survey forms indicated that some individuals stopped fishing in the area after initiation of surveying activities. Surveyors may have been perceived as being “eyes on the reef” and this alone may have provided enough of an incentive for the cessation of fishing by some individuals, who either did not want to participate, or were engaged in fishing activities that would have been perceived as unsustainable by their peers. These results point to the value of community efforts in increasing compliance with formal rules and regulations, as well as informal social mores that govern interactions with other community members and natural resources. Understanding the social, cultural, economic, and ecological determinants of compliance is critical as enforcement is a key determinant of long-term durability of conservation actions [110,111].

Third, our research also has implications for management by governmental agencies, as they relate to data reporting, the total value of coastal fisheries, and developing place-based rules and regulations governing harvest. Our creel surveys revealed a substantially higher level of catch than the state commercial and marine recreational surveys, which largely target different users (Fig 3). The catch recorded through the creel survey in Kīholo Bay (7,353 kg) was higher than the average reported commercial catch (6,218 ± 1593 kg yr^-1^, 2009–13), despite the fact that the area covered by the creel survey was 1/78^th^ of the commercial reporting block (Fig 3B). These findings are consistent with other creel surveys around the state, which strongly suggest that the non-commercial and non-reported reef fishing effort may be far greater than reported commercial landings [18,46,84]. Beyond differences in the amount of catch, the creel survey effort reveals a higher diversity of fishing methods and gear types than those reported commercially. As state statistics are often used as the basis for management decisions, this study reveals the need for more accurate statewide estimates of effort and production in order to better inform coastal fisheries management and planning efforts. As the spatial coverage of creel survey efforts increases, management can increasingly engage these catch baselines, together with ecological surveys and social data on the social, economic, cultural, and nutritional value of the fishery to better inform the development of rules and regulations. Engaging fishers in the production of this information can also take pressure off of overburdened governmental agencies as resource users become more engaged in fishery assessment and sustainable management [30,112].

## Conclusion

Interdisciplinary, social-ecological systems approaches are necessary to understand the full suite of factors that influence the connections between people and nature. In this study, we documented the large-scale benefits that this small-scale fishery provides to communities through a participatory approach that is transferable to other geographies. As this resource-dependent community copes with socioeconomic, cultural, and environmental changes, the interdisciplinary partnership supporting this assessment will continue to generate accurate information and establish trusting relationships with resource users, which are both necessary for successful stewardship. This participatory research uncovers several important factors that influence the linked health of this community and the environment in this small-scale fishery. Just as the ecological health of the fishery affects the ability of harvesters to access and benefit from the resources, social attributes of resource users, including their social networks, motivations, and harvesting method, mediate how the broader community benefits from the resource. Ultimately, this approach and other similar community-based conservation programs need to identify and implement interventions that can increase the flow of benefits to community members, while protecting the key ecological processes that are necessary to support these social, cultural, and economic services in the long-term.

## Supporting Information

S1 Dataset Analytical methods for catch and effort calculations. (PDF)

S2 Dataset All data used in tables and figures for this study. This file serves as the official public data repository for this publication.
(PDF)

S1 Table Hawaii MPA Biomass. Biomass at different marine protected areas (MPA) within the West Hawaii Regional Fishery Management Area (WHRFMA). Apex_bio stands for apex predator biomass (without sharks), H_bio stands for herbivore biomass, P_bio stands for piscivores biomass, S_bio stands for secondary consumer biomass, Z_bio stands for planktivores, and SHRK_bio stands for shark biomass. The units here are grams per meter squared.
(PDF)

S2 Table Fishing Effort Survey. (PDF)

S3 Table Fish Flow Survey. (PDF)

S4 Table Daily Fishing Effort. Average daily fishing effort (gear-hour/day) and number of survey days and days for each quarter.
(PDF)

S5 Table Total Fishing Effort. Expanded fishing effort (in gear-hour) and total fishing effort for each gear type.
(PDF)

S6 Table CPUE Summary. Summary of CPUE (kg/gear-hour) for each gear type between May 2012 –April 2013.
(PDF)

S7 Table Annual Expanded Catch. Annual expected catch (kg) for each gear type.
(PDF)

S8 Table Species Edible Weight. Species-specific estimates for edible weight conversions from live weight, provided by local fishermen’s traditional ecological knowledge.
(PDF)

S9 Table Kīholo Bay Catch and Commercial Reported Catch. Creel survey and expanded catch from Kīholo Bay, by gear type and trophic group, compared to DLNR reported catch for 2009–2013 for reporting block 102, where Kīholo is contained. All values reported in kilograms. These gear types are grouped by fishing methods in manuscript figures (line fishing includes rod and reel as well as handpole for Kiholo Bay; handline is grouped under line fishing for DLNR commercial data).
(PDF)

S10 Table Commercial Reported Catch in West Hawai‘i. DLNR reported catch for 2009–2013 for reporting block 102, with catch value, and fraction of annual catch and annual value represented by each species.
(PDF)
