# Supplementary material for: From Reef to Table: Social and Ecological Factors Affecting Coral Reef Fisheries, Artisanal Seafood Supply Chains, and Seafood Security
Source: PLoS One. 2015 Aug 5;10(8):e0123856. doi: 10.1371/journal.pone.0123856 (PMC4526684; doi:10.1371/journal.pone.0123856)
Supplement: S1 Dataset — (PDF) [file pone.0123856.s001.pdf]

S1 Dataset. Analytical methods for catch and effort calculations

Fishing effort estimation

Daily mean fishing effort for each gear type per quarter was estimated using equation 1

$$\text{Emean} = \frac{\sum_{i=1}^{d}\sum_{j=1}^{N_i} E_{ij}}{d} \quad \text{(eq. 1)}$$

where d was the number of days surveyed for each quarter, N was the number of fishers observed on day i, and Eij is the observed fishing effort of fisher j on day i, where i=1…d, j=1….Ni. Total fishing effort, E, was estimated by multiplying the mean fishing effort (Emean) with total number of days for each quarter.

Catch estimation

Total catch was estimated as a product of the total fishing effort and CPUE for each gear type per quarter. Trophic group composition for each gear type was calculated by multiplying each group's proportion to the corresponding gear's expanded catch.

Catch per unit effort (CPUE) estimation

Average catch per unit effort (CPUE) for each gear type was estimated using the following equation 2

$$U = \frac{\sum_{i=1}^{d}\sum_{j=1}^{n_i} \frac{c_{ij}}{E_{ij}}}{\sum_{i=1}^{d} n_i} \quad \text{........ (eq. 2)}$$

where ni was the number of interviews conducted on day i and cij was catch (in kg) of fisher j on day i, where i = 1…d and j = 1…ni.

Error Propagation

We calculated the standard deviation for the year's total expanded catch for each gear type (line fishing, thrownet, spear, and other) by taking a square root of the sum of the variances of each quarter's total expanded catch for that particular gear. Standard error for the annual total expanded catch for each gear type was then estimated by dividing the standard deviation by a square root of 4 (i.e., the total number of quarterly estimates which formed the annual expansion). Standard deviation on the total expanded catch for the year for all gear combined was determined by performing a standard error propagation analysis with the standard deviations for annual expanded catches for each gear type. The standard error then on the total expanded catch for the year for all gear combined was estimated by dividing the standard deviation by the square root of the 4 quarters of data in the dataset.
