# Supplementary material for: From Reef to Table: Social and Ecological Factors Affecting Coral Reef Fisheries, Artisanal Seafood Supply Chains, and Seafood Security
Source: PLoS One. 2015 Aug 5;10(8):e0123856. doi: 10.1371/journal.pone.0123856 (PMC4526684; doi:10.1371/journal.pone.0123856)
Supplement: S2 Dataset — This file serves as the official public data repository for this publication. (PDF) [file pone.0123856.s002.pdf]

For Public Data Repository: PONE-D-14-49291

TITLE: From reef to table: social and ecological factors affecting coral reef fisheries, artisanal seafood supply chains, and seafood security

AUTHORS: John N. Kittinger[1,2], Lida T. Teneva[1], Haruko Koike[3], Kostantinos A. Stamoulis[3], Daniela S. Kittinger[2], Kirsten L. L. Oleson[4], Eric Conklin[5], Mahana Gomes[6], Bart Wilcox[6], Alan M. Friedlander[3,7]

					Expanded Total Biomass by Trophic Group (kg)				
Structure	*Area (km^2)*	*# Survey Sites*	*Average biomass ($g\ m^{-2}$)*	*±SE*	*Apex Predators*	*Herbivores*	*Secondary Consumers*	*Planktivores*	*Total*
**Rock/Boulder**	1.15	58	25.3	2.8	1,418	13,310	11,194	3,180	29,103
**Pavement with Sand Channels**	0.44	36	22.9	3.6	0	3,611	5,525	979	10,115
**Aggregate Reef**	0.25	18	36.3	5.3	120	2,405	5,944	650	9,118
**Sand**	0.71	N/A	N/A	N/A	N/A	N/A	N/A	N/A	**N/A**
**Total**	2.55	112			1,538	19,326	22,663	4,810	**48,337**

Table 1. Area, number of survey sites, and average biomass for each habitat structure category surveyed in Kīholo Bay. Sand habitat was not surveyed and not included in this table, though it makes up an additional 0.71 km^2 of habitat area (28%) in the bay. SE is standard error of the mean for transect biomass values in each category. Average biomass was expanded to the total area of each

habitat structure category by trophic group, resulting in an estimate of total standing stock of resource fishes.

Fishing method	***Gear Type***	***Fishing Hours***	***CPUE***		***N***
			Mean	***COV***	
**Line Fishing**	Hand pole	2745	7.5	0.9	21
**Line Fishing**	Rod and reel	2259	6.2	0.1	17
**Thrownet**	Thrownet	1434	3.9	0.09	43
**Spear**	3-prong; speargun	799	2.2	0.05	15
**Other**	Limpet (Opihi) Gleaning	460	1.3	0.06	6
**Other**	Crabbing	146	0.4	0.02	3
**Other**	Aquarium	84	0.2	0.01	1
**Other**	Other	**88**	**0.4**	**0.02**	**1**

Table 2. Dominant fishing methods, total effort (fishing hours), and catch per unit effort (CPUE in kg day^{-1}) with means and coefficient of variation (COV) from the one-year creel survey at Kīholo Bay. Number of observed events is also included. Line

fishing includes hand pole, rod-and-reel, and flyfishing. Spear includes 3-prong pole spear and band-powered speargun. Other methods include limpet (*‘opihi*, in Hawaiian) gleaning, crabbing, aquarium fishing.

This study, Kīholo Bay, 2012-2013	*Apex predators*	*Secondary Consumer*	*Herbivore*	*Planktivore*	*Other (including invertebrates)*	*Total by gear type*	*% of total by gear type*
Line Fishing	843	284	20	1891	0	3038	41.3
Thrownet	0	159	2279	16	70	2524	34.3
Spear	0	1038	297	3	0	1338	18.2
Other	0	102	284	0	67	453	6.2
**Total by trophic group**	**843**	**1583**	**2880**	**1909**	**137**	***7353***	
**% of total by trophic group**	**11**	**22**	**39**	**26**	**2**		

Table 3. Total reconstructed catch (in kilograms) for May 1, 2012 – April 30, 2013 for Kīholo Bay by gear type and trophic group.

Year	*Total catch (kg)*	*Total value ($)*	‘ōpelu (*Decapterus* spp) *fraction of catch (%)*	‘ōpelu (*Decapterus* spp) *fraction of value (%)*
**2009**	4,246	48,483	51	44
**2010**	6,859	78,794	56	49
**2011**	8,380	100,538	48	40
**2012**	6,448	75,992	62	53
**2013**	5,159	54,054	71	68
**Mean**	6,218	71,572	58	51
**± SE**	± 712	± 4,184	± 2	± 2

Table 4. Commercial catch (kg) reported to DAR 2009-2013 for the commercial reporting block surrounding Kīholo, with mean and standard error (SE). Total value was computed based on species-level market prices obtained by The Nature Conservancy through market surveys (unpubl.). ‘Opelu (mackerel scad, *Decapterus* spp) fraction of volume and value is also listed.

Trophic Group	*Weight (kg)*	*Fraction of total reported Catch (%)*	*Weight (kg)*	*Fraction of total reported catch that contain end use data (%)*	*Given Away (kg)*	*Kept (kg)*	*Sold (kg)*
Apex Predators	21.1	4	6.6	1.6	0	1.1	0.5
Secondary Consumers	415.3	35.6	114.6	33.5	19.4	8.4	0
Herbivores	404.7	34.7	155.1	32	5.1	30.1	2.5
Planktivores	284.7	24.4	128.5	31.2	8.2	17.5	5.5
Other	15.1	1.3	6.8	1.7	0.8	0.8	0
			**Breakdown by end-use:**		33.50%	58%	8.50%

Table 5. Weight of total reported catch and reported catch with post-landings disposition information, by trophic group, from Kīholo Bay surveys.

Trophic Group	*Economic Value based on MP*	*Fraction of total economic value (%)*	*Nutritional value (no. meals)*	*Fraction of meals (%)*
Apex Predators	$ 12,447	16%	3,321	11%
Secondary Consumers	$ 18,889	24%	6,114	20%
Herbivores	$ 29,247	37%	10,945	36%
Planktivores	$ 17,849	23%	10,107	33%
TOTAL	$ 78,432	100%	30,487	100%

Table 6. Economic and nutritional value of total reconstructed catch for Kīholo Bay (May 1, 2012 – April 30, 2013). MP is market price. Expanded catch by species was paired with market value (TNC, unpublished data). Number of meals assumes 6-ounce servings. Fraction of meals is essentially the percentage of meals delivered by each trophic group for this study.

Data for Figure 2:

MPA	Apex predator biomass (g/m2)	Herbivore biomass (g/m2)	Secondary consumers biomass (g/m2)	Planktivore biomass (g/m2)	Total biomass (g/m2)	Standard Error
NWHI	150.49	115.29	16.63	4.60	287.01	17.73
Kahoolawe	18.69	36.83	32.49	18.96	106.97	12.32
MPA	2.02	31.84	16.20	1.66	51.73	11.26
Open	0.32	15.87	9.42	1.94	27.56	1.40
Kiholo Bay	0.71	10.16	12.87	2.56	26.30	2.06
Notes:						
Kiholo Bay estimates based on 2012 dataset (N=112) to match CREEL data						
Reference sites based on meta-fish database - broader time range						
Sharks included as APEX predators						
Piscivores lumped with						

secondary predators					

Data for Figure 3:

Total Estimated Standing Stock of Resource Fish

Apex Predators (kg)	*Herbivores (kg)*	*Secondary Consumers (kg)*	*Planktivores (kg)*	*Total (kg)*
1,538	19,326	22,663	4,810	**48,337**

This study, Kīholo Bay, 2012-2013	*Apex predators*	*Secondary Consumer*	*Herbivore*	*Planktivore*	*Other (including invertebrates)*	*Total by gear type*	*% of total by gear type*
Line Fishing	843	284	20	1891	0	3038	41.3
Thrownet	0	159	2279	16	70	2524	34.3
Spear	0	1038	297	3	0	1338	18.2
Other	0	102	284	0	67	453	6.2
**Total by trophic group**	**843**	**1583**	**2880**	**1909**	**137**	***7353***	

**% of total by trophic group**	**11**	**22**	**39**	**26**	**2**

Disposition			
Trophic Group	***Given Away (lbs)***	***Kept (lbs)***	***Sold (lbs)***
Apex Predator	0.0	9.7	4.8
Secondary consumer	176.2	76.5	0.0
Herbivore	46.2	273.2	22.5
Planktivore	74.8	158.9	49.6
Other	7.5	7.5	0.0

Data for Figure 4:

	DAR Area 102 total reported catch (in thousands of kg)	***Standard Error***	***Kiholo Bay reconstructed catch 5/2012-4/2013 (in thousands of kg)***	***Standard Error***
Line	3.596	0.899365696	3.038	0.6723

Fishing				
Thrownet	2.272	0.79450975	2.524	0.90935
Spear	0.303	0.130370398	1.339	0.5494
Other	0.047	0.04277096	0.452	0.37985

Data for Figure 5:

Excel name	Distribution Location	Counts for District	Counts for Town	Longitude	Latitude	Number of Distribution Events	Number of Keep Distr Events	Number of Give Away Distr Events
GIS name	Location			longitude	latitude	Dst_Ev	DstEvKP	DstEvGA
	Hamakua	Hamakua	Yes - * a	-155.55	20	2	2	0
	Hilo	Hilo	Hilo	-155.090	19.730	8	4	1
	Honoka'a	Hamakua	Honoka'a	-155.473	20.083	3	1	2
	Kalaoa	North Kona	Kalaoa	-155.982	19.729	2	1	1
	Kawaihae	South Kohala	Kawaihae	-155.827	20.035	1	1	0
	Kealakehe	North Kona	Kealakehe	-155.992	19.670	1	1	0
	Kiholo	North Kona	Kīholo	-155.924	19.858	10	9	0
	Kohala	North + South Kohala	Yes - * c	-155.794	20.132	9	8	1
	Kona	North Kona	Yes - * b	-155.996	19.641	23	18	5
	Pu'uanahulu	North Kona	Pu'uanahulu	-155.847	19.818	5	5	0
	Waikoloa	South Kohala	Waikoloa	-155.793	19.941	10	7	3

	Waimea	South Kohala	Waimea	-155.672	20.02	12	8	4
