# Supplementary material for: From Reef to Table: Social and Ecological Factors Affecting Coral Reef Fisheries, Artisanal Seafood Supply Chains, and Seafood Security
Source: PLoS One. 2015 Aug 5;10(8):e0123856. doi: 10.1371/journal.pone.0123856 (PMC4526684; doi:10.1371/journal.pone.0123856)
Supplement: S1 Table — Biomass at different marine protected areas (MPA) within the West Hawaii Regional Fishery Management Area (WHRFMA). Apex_bio stands for apex predator biomass (without sharks), H_bio stands for herbivore biomass, P_bio stands for piscivores biomass, S_bio stands for secondary consumer biomass, Z_bio stands for planktivores, and SHRK_bio stands for shark biomass. The units here are grams per meter squared. (PDF) [file pone.0123856.s003.pdf]

S1 Table.

Biomass at different marine protected areas (MPA) within the West Hawaii Regional Fishery Management Area (WHRFMA). Apex_bio stands for apex predator biomass (without sharks), H_bio stands for herbivore biomass, P_bio stands for piscivores biomass, S_bio stands for secondary consumer biomass, Z_bio stands for planktivores, and SHRK_bio stands for shark biomass. The units here are grams per meter squared.

MPA_name	Mgmt_status	APEX_bio	H_bio	P_bio	S_bio	Z_bio	SHRK_bio	Tot_Bio	Std_Er
Old Kona Airport	Restricted access	0.00	89.03	6.47	15.01	0.10	0.00	110.61	
Old Kona Airport	Partial	1.06	39.25	10.56	12.80	0.73	0.00	64.39	
Kealakekua Bay	Full	6.11	19.18	6.66	22.11	4.23	0.00	58.29	
Lapakahi	Partial	0.59	17.19	6.57	11.01	6.04	0.00	41.41	
Waialea Bay	Partial	1.01	24.66	1.81	1.95	0.21	0.00	29.63	
Kealakekua Bay	Partial	0.16	15.49	3.11	7.66	0.30	4.60	31.32	
Lapakahi	Full	0.59	18.11	2.10	5.57	0.04	0.00	26.42	
**AVERAGE**		1.36	31.84	5.33	10.87	1.66	0.66	51.73	11.26
