# Supplementary material for: From Reef to Table: Social and Ecological Factors Affecting Coral Reef Fisheries, Artisanal Seafood Supply Chains, and Seafood Security
Source: PLoS One. 2015 Aug 5;10(8):e0123856. doi: 10.1371/journal.pone.0123856 (PMC4526684; doi:10.1371/journal.pone.0123856)
Supplement: S2 Table — (PDF) [file pone.0123856.s004.pdf]

S2 Table. Fishing Effort Survey

Fishing Effort Survey **Kīholo Bay, Hawaiʻi Island**

Date: ___________________ **Surveyor Name:**______________________________________

Survey Time Frame (Circle One): 7am-10am 10am-1pm 1pm-4pm 4pm-7pm

Zone (Circle one): **1** **2** **3**

Weather Condition (Circle one): Sunny Rainy Overcast

Wind/Water Condition (Circle one): Calm/Clear Kona Normal Trades Gusty/Rough

Tide Condition (Circle one): Rising Dropping Slack

Event #	Zone	Time start	Time Stop	Gear Type/Activity	Boat-Based? Y/N	# of Gears	# of Fishers	Catch (if ID)	Notes/Observations
1									
2									
3									
4									
5									
6									
7									
8									
9									
10									

Notes:
