# Supplementary material for: From Reef to Table: Social and Ecological Factors Affecting Coral Reef Fisheries, Artisanal Seafood Supply Chains, and Seafood Security
Source: PLoS One. 2015 Aug 5;10(8):e0123856. doi: 10.1371/journal.pone.0123856 (PMC4526684; doi:10.1371/journal.pone.0123856)
Supplement: S3 Table — (PDF) [file pone.0123856.s005.pdf]

S3 Table. Fish Flow Survey

Fishing Intercept Survey Form

Gender: M F Age: ___________

Education level _______________Occupation __________________

Birthplace: __________________ Currently resides: ______________

Ethnicity _________________________Native Hawaiian? Y N

Gear type/ Activity	Species/ Family	# Caught	# (Act/ Est)	Weight (lbs)	Weight (Act/ Est)	Zone	Notes
			A / E		A / E	1<br>2<br>3	
			A / E		A / E	1<br>2<br>3	
			A / E		A / E	1<br>2<br>3	
			A / E		A / E	1<br>2<br>3	
			A / E		A / E	1<br>2<br>3	
			A / E		A / E	1<br>2<br>3	
			A / E		A / E	1<br>2<br>3	
			A / E		A / E	1<br>2<br>3	

TOTALS			
# Gears	# Species	Weight (lbs)	# hours fished

Notes:

Fish Flow data

Event # 1

	%	Check any/all that apply:	LOCATION (use map on following page) Code (for map entry**)	Address, closest cross-street, town, or ahupua'a:	
% KEPT:			1		
% GIVEN AWAY:		☐ Family (outside household)	2		
		☐ Family (outside household)	3		
		☐ Friends	4		
		☐ Friends	5		
		☐ Specifically for pa'ina	6		
		☐ Other<br>List:	7		
	%	Check any/all that apply:	LOCATION (use map on following page) Code (for map entry**)	Address, closest cross-street, town or ahupua'a:	PRICE (or $$ value)
% FOR SALE:		☐ Friends/family	8		
		☐ Friends/family	9		
		☐ Local minimarket/grocer	10		
		☐ Local minimarket/grocer	11		
		☐ Ope n market	12		
		☐ Ope n market	13		
		☐ Foodland	14		
		☐ Distributor	15		
		☐ Traded<br>Who?	16		
		☐ Other (auction, restaurant, etc)<br>List:	17		
% RE-LEASED:		Reason:			
% OTHER:		List:	18		
TOTAL = 100					

** Maps found at end of booklet. One map per event.
