# Supplementary material for: From Reef to Table: Social and Ecological Factors Affecting Coral Reef Fisheries, Artisanal Seafood Supply Chains, and Seafood Security
Source: PLoS One. 2015 Aug 5;10(8):e0123856. doi: 10.1371/journal.pone.0123856 (PMC4526684; doi:10.1371/journal.pone.0123856)
Supplement: S4 Table — Average daily fishing effort (gear-hour/day) and number of survey days and days for each quarter. (PDF) [file pone.0123856.s006.pdf]

S4 Table.

Average daily fishing effort (gear-hour/day) and number of survey days and days for each quarter.

Quarter	Days in each Quarter	Survey Days	HandPole	Rod&Pole	ThrowNet	Spear	Opihi	Crabbing	Other	Aquarium	FlyFishing
1	92	20	0.40	9.44	4.29	2.31	0.70	0.00	0.72	0.77	0.20
2	92	18	26.60	3.96	2.36	4.42	0.00	1.50	0.08	0.00	0.11
3	92	12	1.42	6.52	2.77	0.67	2.42	0.00	0.00	0.00	0.25
4	89	17	0.00	4.49	5.97	0.75	2.44	0.00	0.00	0.00	0.00
