# Supplementary material for: From Reef to Table: Social and Ecological Factors Affecting Coral Reef Fisheries, Artisanal Seafood Supply Chains, and Seafood Security
Source: PLoS One. 2015 Aug 5;10(8):e0123856. doi: 10.1371/journal.pone.0123856 (PMC4526684; doi:10.1371/journal.pone.0123856)
Supplement: S5 Table — Expanded fishing effort (in gear-hour) and total fishing effort for each gear type. (PDF) [file pone.0123856.s007.pdf]

S5 Table.

Expanded fishing effort (in gear-hour) and total fishing effort for each gear type.

Quarter	HandPole	Rod&Pole	ThrowNet	Spear	Opihi	Crabbing	Other	Aquarium	FlyFishing
1	36.80	868.87	394.46	212.75	64.40	0.00	66.13	70.73	18.40
2	2446.94	364.17	217.22	406.33	0.00	138.00	7.67	0.00	10.22
3	130.33	599.92	254.92	61.33	222.33	0.00	0.00	0.00	23.00
4	0.00	399.19	531.38	66.75	217.26	0.00	0.00	0.00	0.00
Total	2614.08	2232.14	1397.98	747.17	504.00	138.00	73.80	70.73	51.62
