# Supplementary material for: From Reef to Table: Social and Ecological Factors Affecting Coral Reef Fisheries, Artisanal Seafood Supply Chains, and Seafood Security
Source: PLoS One. 2015 Aug 5;10(8):e0123856. doi: 10.1371/journal.pone.0123856 (PMC4526684; doi:10.1371/journal.pone.0123856)
Supplement: S6 Table — Summary of CPUE (kg/gear-hour) for each gear type between May 2012 –April 2013. (PDF) [file pone.0123856.s008.pdf]

S6 Table.

Summary of CPUE (kg/gear-hour) for each gear type between May 2012 – April 2013.

Gear Type	# of Interview	Q1	Q2	Q3	Q4	Yearly Mean
Spear	15	1.32	1.24	5.22	0.91	1.79
Throw Net	43	1.17	1.03	2.64	2.21	1.81
Crabbing	3	NA	0.72	NA	NA	0.72
Rod&Pole	17	0.95	1.10	0.04	NA	0.89
Opihi	6	NA	NA	1.24	0.15	0.69
Hand Pole	21	0.00	0.41	0.50	NA	0.40
Other	1	0.03	NA	NA	NA	0.03
Aquarium	1	0.00	NA	NA	NA	0.00
