# Supplementary material for: From Reef to Table: Social and Ecological Factors Affecting Coral Reef Fisheries, Artisanal Seafood Supply Chains, and Seafood Security
Source: PLoS One. 2015 Aug 5;10(8):e0123856. doi: 10.1371/journal.pone.0123856 (PMC4526684; doi:10.1371/journal.pone.0123856)
Supplement: S7 Table — Annual expected catch (kg) for each gear type. (PDF) [file pone.0123856.s009.pdf]

S7 Table.

Annual expected catch (kg) for each gear type.

Expanded Catch	HandPole	Rod &Pole	ThrowNet	Spear	Opihi	Crabbing	Other
Yearly Mean CPUE	0.40	0.89	1.81	1.79	0.69	0.72	0.03
Q1	14.69	776.40	712.12	381.18	44.79	0.00	2.10
Q2	976.72	325.41	392.15	728.02	0.00	99.55	0.24
Q3	52.02	536.07	460.20	109.89	154.63	0.00	0.00
Q4	0.00	356.71	959.30	119.59	151.11	0.00	0.00
Total Catch (lbs)	1043.44	1994.59	2523.77	1338.69	350.53	100.27	2.35
