# Supplementary material for: From Reef to Table: Social and Ecological Factors Affecting Coral Reef Fisheries, Artisanal Seafood Supply Chains, and Seafood Security
Source: PLoS One. 2015 Aug 5;10(8):e0123856. doi: 10.1371/journal.pone.0123856 (PMC4526684; doi:10.1371/journal.pone.0123856)
Supplement: S8 Table — Species-specific estimates for edible weight conversions from live weight, provided by local fishermen’s traditional ecological knowledge. (PDF) [file pone.0123856.s010.pdf]

# S8 Table.

Species-specific estimates for edible weight conversions from live weight, provided by local fishermen's traditional ecological knowledge.

| Hawaiian Name | English Name | Latin Name | Edible Weight Fraction |
|---|---|---|---|
| Aama | Rock crab | Grapsus tenuicrustatus | 0.50 |
| Aholehole | Hawaiian Flagtail | Kuhlia sandvicensis | 0.67 |
| Aala.ihi | Bluestripe squirrelfish | Sargocentron tiere | 0.67 |
| Halalu | Bigeye scad | Selar crumenophthalmus | 0.90 |
| Hinalea | | | 0.67 |
| Kala | Bluespine unicornfish | Naso unicornis | 0.80 |
| Kole | Goldenring surgeonfish | Ctenochaetus strigosus | 0.67 |
| Kumu | Whitesaddle goatfish | Parupeneus porphyreus | 0.67 |
| Kupipi | Blackspot sergeant | Abudefduf sordidus | 0.67 |
| Maiko | Bluelined Surgeonfish | Acanthurus nigroris | 0.67 |
| Manini | Convict Tang | Acanthurus triostegus | 0.67 |
| Mempachi | Soldierfish | Myripristis spp | 0.67 |
| Moana.Kali | Gold-saddle goatfish | Parupeneus cyclostomus | 0.67 |
| Moano | Manybar Goatfish | Parupeneus multifasciatus | 0.67 |
| Mullet | Mullet | Neomyxus leuciscus | 0.67 |
| Nainai | Orangeband Surgeonfish | Acanthurus olivaceus | 0.67 |
| Nenui | Chub | Kyphosus Vaigiensis | 0.67 |
| Oama | baby Orange goatfish | Mulloidichthys pfluegeri | 0.90 |
| Omamalei | Orangespine Unicornfish | Naso lituratus | 0.80 |
| Omilu | Bluefin Trevally | Caranx melampygus | 0.67 |
| Opihi | Limpet | Tectura scutum | 0.50 |
| Pakukui | Achilles Tang | Acanthurus achilles | 0.67 |
| Papio | Juvenile Trevally (ulua) (<10lbs) | Caranx ignobilis | 0.67 |
| Roi | Peacock grouper | Cephalopholis argus | 0.67 |
| Taape | Bluestripe snapper | Lutjanus kasmira | 0.67 |
| Toao | Blacktail snapper | Lutjanus fulvus | 0.67 |
| Uhu | Bullethead parrotfish | Chlorurus spilurus | 0.67 |
| Ulua | Giant Trevally (Big Jack) | Caranx ignobilis | 0.67 |
| Uo.Uoa | Sharpnose mullet | Neomyxus leuciscus | 0.67 |
| Weke | Goatfish | Mulloidichthys spp | 0.67 |
