# Supplementary material for: From Reef to Table: Social and Ecological Factors Affecting Coral Reef Fisheries, Artisanal Seafood Supply Chains, and Seafood Security
Source: PLoS One. 2015 Aug 5;10(8):e0123856. doi: 10.1371/journal.pone.0123856 (PMC4526684; doi:10.1371/journal.pone.0123856)
Supplement: S9 Table — Creel survey and expanded catch from Kīholo Bay, by gear type and trophic group, compared to DLNR reported catch for 2009–2013 for reporting block 102, where Kīholo is contained. All values reported in kilograms. These gear types are grouped by fishing methods in manuscript figures (line fishing includes rod and reel as well as handpole for Kiholo Bay; handline is grouped under line fishing for DLNR commercial data). (PDF) [file pone.0123856.s011.pdf]

S9 Table.

Creel survey and expanded catch from Kīholo Bay, by gear type and trophic group, compared to DLNR reported catch for 2009-2013 for reporting block 102, where Kīholo is contained. All values reported in kilograms. These gear types are grouped by fishing methods in manuscript figures (line fishing includes rod and reel as well as handpole for Kiholo Bay; handline is grouped under line fishing for DLNR commercial data).

This study, Kīholo Bay, 2012-2013	***Apex***	***Secondary Consumer***	***Herbivore***	***Planktivore***	***Other (including invertebrates)***	***Total by gear type***
Line Fishing	842.6	284.2	20.2	1891	0	3038
Thrownet	0	158.8	2279.1	15.75	70.1	2523.8
Spear	0	1038.1	297.4	3.2	0	1338.7
Other	0	101.9	283.5	0	67.1	452.4
**Total:**	**842.6**	**1583**	**2880.2**	**1909.9**	**137.2**	**7352.9**
DLNR, 2009	***Apex***	***Secondary Consumer***	***Herbivore***	***Planktivore***	***Other (including invertebrates)***	
Line Fishing	1,223	483	0	2,152	0	3858
Thrownet	0	326	0	0	0	326
Spear/Dive	0	36	10	0	0	46
Troll	16	0	0	0	0	16
**Totals:**	**1,239**	**845**	**10**	**2,152**	**0**	**4246**
DLNR, 2010	***Apex***	***Secondary Consumer***	***Herbivore***	***Planktivore***	***Other (including invertebrates)***	
Line Fishing	1,899	382	45	3,951	0	6277
Thrownet	0	327	0	0	0	327
Spear/Dive	0	255	0	0	0	255
Troll	0	0	0	0	0	0
**Totals:**	**1,899**	**964**	**45**	**3,951**	**0**	**6859**
DLNR, 2011	***Apex***	***Secondary Consumer***	***Herbivore***	***Planktivore***	***Other (including invertebrates)***	
Line Fishing	3,102	393	14	1,060	0	4569
Thrownet	0	348	0	3,195	0	3543
Spear/Dive	0	268	0	0	0	268
Troll	0	0	0	0	0	0
**Totals:**	**3,102**	**1,009**	**14**	**4,255**	**0**	**8380**
DLNR, 2012	***Apex***	***Secondary Consumer***	***Herbivore***	***Planktivore***	***Other (including invertebrates)***	
Line Fishing	991	308	0	734	0	2033
Thrownet	0	164	0	3,452	0	3616
Spear/Dive	0	687	112	0	0	799
Troll	0	0	0	0	0	0
**Totals:**	**991**	**1,158**	**112**	**4,186**	**0**	**6448**
DLNR, 2013	***Apex***	***Secondary Consumer***	***Herbivore***	***Planktivore***	***Other (including invertebrates)***	
Line Fishing	588	63	19	574	0	1244
Thrownet	0	224	0	3,326	0	3549
Spear/Dive	0	148	0	0	0	148
Troll	217	0	0	0	0	217
**Totals:**	**806**	**434**	**19**	**3,900**	**0**	**5159**
