# Supplementary material for: From Reef to Table: Social and Ecological Factors Affecting Coral Reef Fisheries, Artisanal Seafood Supply Chains, and Seafood Security
Source: PLoS One. 2015 Aug 5;10(8):e0123856. doi: 10.1371/journal.pone.0123856 (PMC4526684; doi:10.1371/journal.pone.0123856)
Supplement: S10 Table — DLNR reported catch for 2009–2013 for reporting block 102, with catch value, and fraction of annual catch and annual value represented by each species. (PDF) [file pone.0123856.s012.pdf]

S10 Table.

DLNR reported catch for 2009-2013 for reporting block 102, with catch value, and fraction of annual catch and annual value represented by each species.

Year	Method	Species	Lbs caught	Kg caught	Trophic Group		% of annual catch	% of annual value
2009	Handline	Opelu	4,744	2,152	Planktivore		50.7	44.1
2009	Handline	Uku	2,065	937	Apex Predator		22.1	28.8
2009	Handline	Menpachi	964	437	Secondary consumer		10.3	15.7
2009	Handline	Kahala	545	247	Apex Predator		5.8	5.6
2009	Handline	Laenihi	92	42	Secondary consumer		1.0	2.6
2009	Handline	Kamanu	46	21	Apex Predator		0.5	N/A
2009	Handline	White ulua	41	19	Apex Predator		0.4	0.6
2009	Handline	Taape	9	4	Secondary consumer		0.1	0.1
2009	Net	Kona crab	718	326	Secondary consumer		7.7	N/A
2009	Spear/Dive	Roi	60	27	Secondary consumer		0.6	1.0
2009	Spear/Dive	Palani	21	10	Herbivore		0.2	0.1
2009	Spear/Dive	Menpachi	20	9	Secondary consumer		0.2	0.3
2009	Troll	Kaku	35	16	Apex Predator		0.4	1.1
		**TOTAL**	**9,360**	**4,246**			**100**	**100**
2010	Handline	Opelu	8,521	3,865	Planktivore		56.4	48.8
2010	Handline	Uku	2,701	1,225	Apex Predator		17.9	23.2
2010	Handline	Kahala	1,166	529	Apex Predator		7.7	7.3
2010	Handline	Laenihi	598	271	Secondary consumer		4.0	10.2
2010	Handline	White ulua	268	122	Apex Predator		1.8	2.4
2010	Handline	Taape	162	73	Secondary consumer		1.1	0.6
2010	Handline	Akule	160	73	Planktivore		1.1	1.1
2010	Handline	Opelu kala	99	45	Herbivore		0.7	0.3
2010	Handline	Kamanu	52	24	Apex Predator		0.3	N/A
2010	Handline	Weke nono	49	22	Secondary consumer		0.3	0.4
2010	Handline	Aawa	20	9	Secondary consumer		0.1	0.1
2010	Handline	Weke ula	20	9	Planktivore		0.1	0.2
2010	Handline	Toau	13	6	Secondary consumer		0.1	0.1
2010	Handline	Moana kale	9	4	Planktivore		0.1	0.2
2010	Net	Kona crab	721	327	Secondary consumer		4.8	N/A
2010	Spear/Dive	Day tako	402	182	Secondary consumer		2.7	3.5
2010	Spear/Dive	Menpachi	160	73	Secondary consumer		1.1	1.6
		**TOTAL**	**15,121**	**6,859**			**100**	**100**
2011	Handline	Uku	6,524	2,959	Apex Predator		35.3	43.9
2011	Handline	Opelu	1,772	804	Planktivore		9.6	7.9
2011	Handline	Menpachi	591	268	Secondary consumer		3.2	4.6
2011	Handline	Akule	565	256	Planktivore		3.1	2.9
2011	Handline	Kahala	315	143	Apex Predator		1.7	1.6
2011	Handline	Laenihi	230	104	Secondary consumer		1.2	3.1
2011	Handline	Taape	45	20	Secondary consumer		0.2	0.1
2011	Handline	Opelu kala	30	14	Herbivore		0.2	0.1
2011	Net	Opelu	7,043	3,195	Planktivore		38.1	31.6
2011	Net	Kona crab	768	348	Secondary consumer		4.2	N/A
2011	Spear/Dive	Day tako	591	268	Secondary consumer		3.2	4.1
		**TOTAL**	**18,474**	**8,380**			**100**	**100**

2012	Handline	Uku	1,997	906	Apex Predator		14.0	17.8
2012	Handline	Opelu	1,265	574	Planktivore		8.9	7.5
2012	Handline	Laenihi	386	175	Secondary consumer		2.7	6.9
2012	Handline	Akule	327	148	Planktivore		2.3	2.3
2012	Handline	Kahala	160	73	Apex Predator		1.1	1.0
2012	Handline	Weke nono	125	57	Secondary consumer		0.9	1.0
2012	Handline	Menpachi	110	50	Secondary consumer		0.8	1.1
2012	Handline	Weke ula	39	18	Secondary consumer		0.3	0.3
2012	Handline	Kamanu	28	13	Apex Predator		0.2	N/A
2012	Handline	Aweoweo	26	12	Planktivore		0.2	0.2
2012	Handline	Taape	18	8	Secondary consumer		0.1	0.1
2012	Net	Opelu	7,610	3,452	Planktivore		53.5	45.2
2012	Net	Kona crab	362	164	Secondary consumer		2.5	N/A
2012	Spear/Dive	Day tako	1,373	623	Secondary consumer		9.7	12.6
2012	Spear/Dive	Uhu parrot-misc.	248	112	Herbivore		1.7	2.2
2012	Spear/Dive	Menpachi	70	32	Secondary consumer		0.5	0.7
2012	Spear/Dive	Kumu	40	18	Secondary consumer		0.3	0.8
2012	Spear/Dive	Mu	31	14	Secondary consumer		0.2	0.3
		**TOTAL**	**14,215**	**6,448**			**100**	**100**
2013	Handline	Opelu	767	348	Planktivore		6.7	6.4
2013	Handline	Uku	682	309	Apex Predator		6.0	8.5
2013	Handline	Kahala	615	279	Apex Predator		5.4	5.6
2013	Handline	Akule	499	226	Planktivore		4.4	4.8
2013	Handline	Laenihi	107	49	Secondary consumer		0.9	2.7
2013	Handline	Opelu kala	42	19	Herbivore		0.4	0.2
2013	Handline	Weke nono	32	14	Secondary consumer		0.3	0.4
2013	Net	Opelu	7,332	3,326	Planktivore		64.5	61.2
2013	Net	Kona crab	493	224	Secondary consumer		4.3	N/A
2013	Spear/Dive	Day tako	326	148	Secondary consumer		2.9	4.2
2013	Troll	Uku	479	217	Apex Predator		4.2	6.0
			**11,374**	**5,159**				
